# Effect of Rosifoliol/Agarospirol and 7α-Hydroxysandaracopimaric Acid from *Salvia elegans* Vahl on Local Inflammation and LPS-Activated RAW-Blue Cells

**DOI:** 10.3390/molecules31183180

**Published:** 2026-09-10

**Authors:** Maribel Herrera-Ruiz, Manasés González-Cortazar, Verónica Sánchez-Sánchez, Gabriela Belen Martínez-Hernández, Alejandro Zamilpa, Yury Maritza Zapata-Lopera, Enrique Jiménez-Ferrer

**Affiliations:** Centro de Investigación Biomédica del Sur, Instituto Mexicano del Seguro Social (IMSS), Xochitepec 62790, Morelos, Mexico; cibis_herj@yahoo.com.mx (M.H.-R.); gmanases2000@gmail.com (M.G.-C.); fdg_105@hotmail.com (V.S.-S.); gabmh89@gmail.com (G.B.M.-H.); yurymzapata@gmail.com (Y.M.Z.-L.)

**Keywords:** Lamiaceae, 7α-hydroxysandaracopimaric acid, rosifoliol, agaraspirol, flavonoids, RAW-Blue^TM^, NF-κB

## Abstract

*Salvia elegans* Vahl. is used to treat inflammatory conditions; however, its direct anti-inflammatory activity has been scarcely investigated. This study evaluated the ethyl acetate extract (SeAcOEt) in vivo and in vitro, using the 12-O-tetradecanoylphorbol-13-acetate (TPA)-induced mouse ear edema model, and the effects of selected isolated metabolites on inflammatory signaling by measuring NF-κB/AP-1-dependent SEAP reporter activity in LPS-stimulated RAW-Blue™ macrophages. Phytochemical characterization was performed using thin-layer chromatography, column chromatography, high-performance liquid chromatography (HPLC), mass spectrometry, and nuclear magnetic resonance (NMR). SeAcOEt yielded the following three fractions: SeFA, containing the terpene mixture rosifoliol (1)/agarospirol (2); SeFB, ursolic acid (3) and 7α-hydroxysandaracopimaric acid (4); and SeFC, jaceosidin (5) and isosakuranetin-5-O-rutinoside (6). SeAcOEt inhibited TPA-induced ear edema by 87.3%, whereas SeFB showed the highest activity (97.4%), comparable to indomethacin. The terpene mixture and 7α-hydroxysandaracopimaric acid reduced NF-κB/AP-1-dependent SEAP reporter activity in LPS-stimulated RAW-Blue™ macrophages. The anti-inflammatory activity of *S. elegans* could be associated with the combined contribution of phytochemicals enriched in the bioactive fraction SeFB rather than with a single active constituent. This study expands the phytochemical knowledge of *S. elegans* by reporting 7α-hydroxysandaracopimaric acid and provides pharmacological evidence supporting the traditional use of this species for inflammatory conditions.

## 1. Introduction

Inflammation is a protective host response to infection or tissue injury, involving coordinated processes such as vasodilation and immune cell recruitment to the injury site. However, dysregulated inflammatory responses contribute to many acute and chronic diseases. Among key inflammation regulators, nuclear factor kappa B (NF-κB) plays a central role by controlling the expression of multiple pro-inflammatory genes in both innate and adaptive immune cells [1]. Consequently, cell-based assays targeting NF-κB signaling have become valuable pharmacological tools for identifying novel anti-inflammatory agents. RAW-Blue™ macrophages, derived from RAW 264.7 cell line, constitutively express a secreted embryonic alkaline phosphatase (SEAP) reporter gene under the control of NF-κB and AP-1 transcription factors. After stimulation of pattern recognition receptors, including Toll-like receptors (TLRs), these transcription factors activate SEAP secretion [2].

Medicinal plants remain an important source of bioactive compounds with anti-inflammatory potential. Among them are species of the genus Salvia, one of the most extensive in the Lamiaceae family, comprising more than 900 species worldwide. Species in this genus have diverse medicinal uses, including sedative, hypnotic, antinociceptive, acetylcholinesterase inhibitor, anticonvulsant, antipyretic, anti-inflammatory, smooth muscle relaxant, neuroprotective, and antihypertensive activities [3]. Sufficient pharmacological information exists on the anti-inflammatory properties of the Salvia genus [4,5], which has been recognized as a rich source of terpenoids, flavonoids, phenolic acids, and other specialized metabolites with pharmacological activity [6,7]. Several studies have shown that species of this genus have anti-inflammatory activity in macrophage cultures. One example is *S. miltiorrhiza;* reports of its anti-inflammatory activity are extensive, demonstrating the plant’s ability to reduce inflammation across various models and pharmacological targets [8]. Notably, a study reported that the plant extract and certain terpenes (tanshinone IIA, miltirone, and cryptotanshinone) exert an anti-inflammatory effect by reducing nitric oxide (NO) release in lipopolysaccharide (LPS)-stimulated RAW264.7 macrophages [9]. From the medicinal species *S. multicaulis*—native to Central and Eastern Asia and traditionally used for wound healing, as an anti-inflammatory, and for pain relief—a terpenoid named salvimulticanol was isolated; this compound is capable of suppressing LPS-induced NO release in RAW264.7 macrophages in a concentration-dependent manner [10]. Another terpenoid, salviplenoid A, isolated from S. plebeian, a medicinal plant used in China to treat inflammation, reduced levels of NO, iNOS, TNF-α, and COX-2 in LPS-stimulated RAW264.7 macrophages, an effect associated with inhibition of NF-κB transcriptional activity [11].

México is one of the principal centers of diversification of the genus, harboring many native species [4,12]. For example, *Salvia elegans* Vahl., also known as “red-flowered myrtle,” “English myrtle,” or jetcho deni (Otomí), is native to Mexico and Central America. This semi-woody perennial herb reaches 1.0 to 1.5 m in height and inhabits temperate climates at elevations of 2280 to 3100 m, commonly found in pine, oak, and mixed forests. It prefers well-drained, moist soils with an acidic pH and variable texture, primarily on hillside slopes. In Mexico, documented distributions include Sonora, Chihuahua, Durango, Michoacán, Hidalgo, the State of Mexico, Morelos, Mexico City, Veracruz, and Oaxaca. *S. elegans* has notable ethnobotanical uses in central Mexico as follows: leaf infusions are taken for abdominal pain, it is used as a mild hypnotic (either by placing sprigs under pillows or as an infusion), root and stem poultices are applied for edema and trauma, and in Puebla, it is employed for gynecological and obstetric purposes such as postpartum recovery and newborn bathing [13]. This plant is traditionally used to treat inflammatory conditions, including traumatic edema [12]. Nevertheless, despite its traditional use, the direct anti-inflammatory activity of *S. elegans* has not been experimentally evaluated using validated pharmacological models.

Previous pharmacological studies have demonstrated antihypertensive activity through inhibition of angiotensin-converting enzyme (ACE) and antagonism of angiotensin II [14,15], as well as anxiolytic and antidepressant effects in behavioral animal models [16,17]. In addition, administration of its ethyl acetate extract and derived fractions reduced pro-inflammatory cytokine concentrations in different organs of mice with diet-induced obesity [18].

Phytochemical studies of *S. elegans* have isolated and identified several secondary metabolites, including terpenoids, flavonoids, phenylpropanoids, and sterols [15,16,17]. Among these metabolites, rosifoliol/agarospirol [19] and isosakuranetin-5-O-rutinoside [17] have been previously reported. However, the metabolites responsible for, or contributing to, the anti-inflammatory activity of *S. elegans* have not yet been identified, and the relationship between its phytochemical composition and anti-inflammatory effects remains poorly understood.

Therefore, this study aimed to characterize the anti-inflammatory activity of the ethyl acetate extract of *S. elegans* (SeAcOEt) by pharmacologically evaluating its bioactive fractions and major isolated metabolites using complementary in vivo and in vitro experimental models. We used the TPA-induced mouse ear edema model to determine the contribution of the extract, fractions, and isolated metabolites to overall anti-inflammatory activity. In addition, the major isolated metabolites, compound 4 (7α-hydroxysandaracopimaric acid) and the rosifoliol/agarospirol mixture (TM; compounds 1/2), were evaluated for their effects on NF-κB/AP-1 signaling by quantifying SEAP reporter activity in LPS-stimulated RAW-Blue™ macrophages.

## 2. Results

### 2.1. Compounds from Fractions SeFA, SeFB and SeFC

Fraction SeFA obtained from the chromatographic fractionation of the ethyl acetate extract (SaAcOE) was analyzed by gas chromatography-mass spectrometry (GC-MS) (see Figure S1) and allowed us to identify that this fraction mainly contains the terpenes called 2-(2R,4aS,8R)-4a,8-dimethyl-2,3,4,4a,5,6,7,8-octahydronaphthalen-2-yl)propan-2-ol, known as rosifoliol (**1**) in 25.2% and 2-(2R,5R,10R)-6,10-dimethylspiro[4.5]dec-6-en-2-yl)propan-2-ol or agarospirol (**2**) at 19.5%—this mixture is called TM. Compounds that had already been previously identified in this species are presented [20].

Chromatographic fractionation of SeFB yielded compound (**3**) as a colorless powder, which was compared by HPLC with a standard ursolic acid sample (UA, **3**). ESI-MS of positive ions revealed quasi-molecular peaks at *m*/*z* 455.8 [M]^+^ for the molecular formula C_30_H_48_O_3_ (*m*/*z* = 456.36) and 395 [M-H_2_O-CO_2_ + H]^+^ for the molecular formula C_29_H_46_ (*m*/*z* = 394.3) (see Figures S2 and S3).

**Figure 1:**
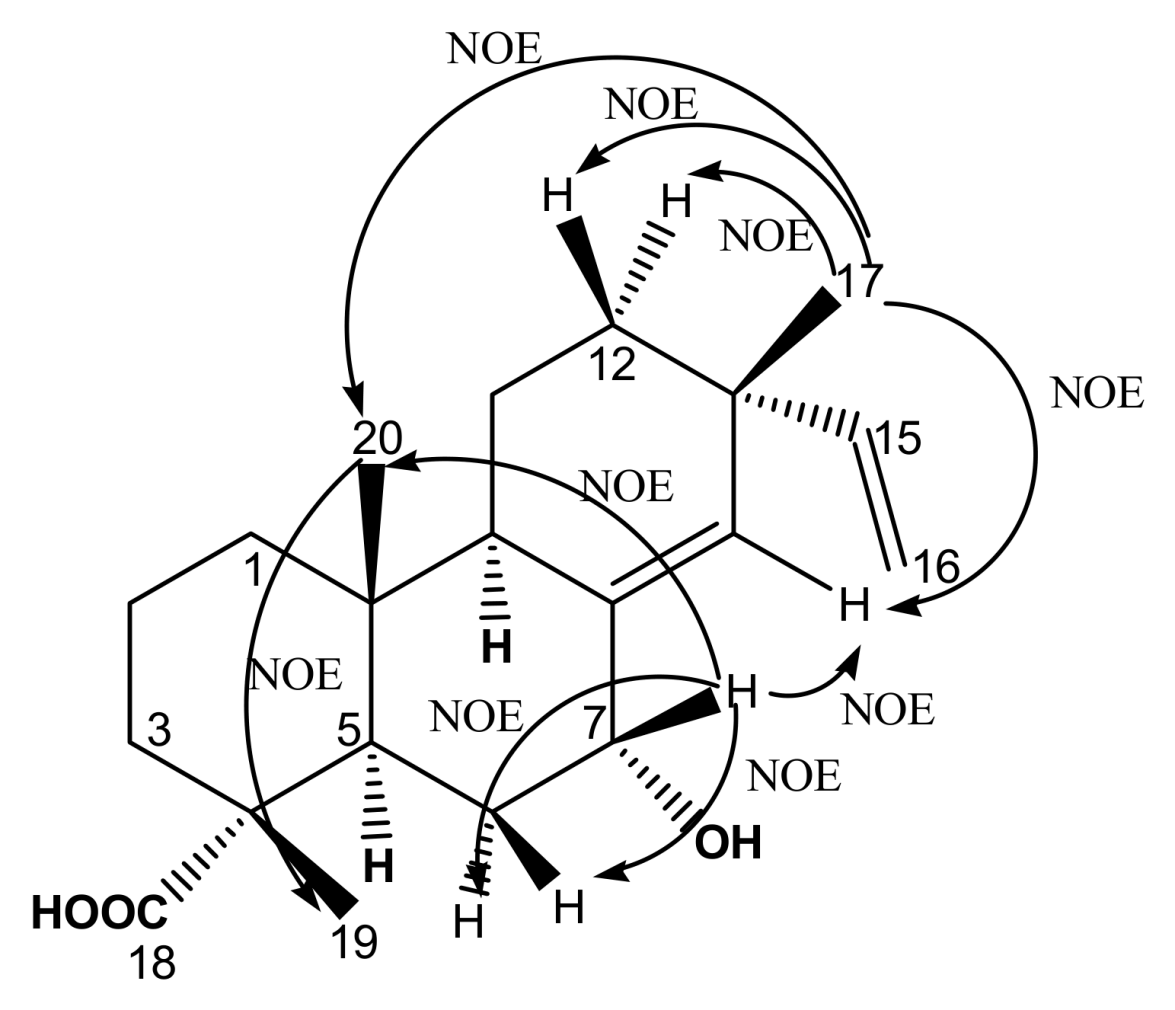
NOESY interactions of compound (**4**).

Compound **4** was a white, amorphous solid (1170 mg), soluble in methanol with a melting point of 91 °C. In the UV light spectrum, the compound showed a λ_max_ 192 nm, characteristic of terpene. The ^13^C NMR spectrum showed twenty signals, which correspond to a diterpene, and according to the DEPT experiment, five are quaternary, five methine, seven methylene and three methyl. The ^1^H NMR spectrum showed the following two signals corresponding to a vinylic ABX system: δ 5.8 (1H, dd, *J* = 10.6, 17.5 Hz), 4.91 (1H, dd, *J* = 1.4, 10.6 Hz) and 4.99 (1H, dd, *J* = 1.4, 17.5 Hz) assigned a H-15, H-16a and H-16b respectively, and a A system [δ 5.51 (H, d,br, *J* = 1.4 Hz) assigned a H-14. According to the HMBC experiment, H-15 (δ 5.8) correlates with three carbon bands at δ 135.1, 38.6 and 26.4, assigned to C-14, C-13 and C-17, respectively. The orientation of the methyl group Me-17 (δH 1.05) in the β position was established by the field interaction observed in the NOESY experiment with the hydrogens of H-20 (δH 0.85), H-12a (δH 1.5), H-12b (δH 1.4) and H-14 (δH 5.5). Additionally, a proton NMR observed an oxygen base at δ 4.1 (dd, *J* = 2.35, 2.82) assigned to H-7 and which correlates in HSQC with carbon at δ 73.86, the orientation of the hydroxyl group in the α position at C-7 and the hydrogen H-7 in the β position (δH 4.10) was evidenced by the NOESY interaction of H-7 observed among H-20 (δH 0.85), H-14 (δH 5.5), H-6a (δH 1.68), and H-6b (δH 1.42) (Figure 1).

According to these spectroscopic data analyses (Table 1) and the comparison with data described in the literature [21], this compound corresponds to a diterpene of isopimaric acid type with a hydroxyl group in position α in C-7, denominated 7α-hydroxy-8(14),15-isopimaradien-18-oic acid, also known as 7α-hydroxysandaracopimaric acid (**4**, 180.7 mg, Figure 1 and Figure 4). This compound has been identified in the bark of *Cryptomeria japonica* and is the first time isolated from *S. elegans*.

**Figure 2:**
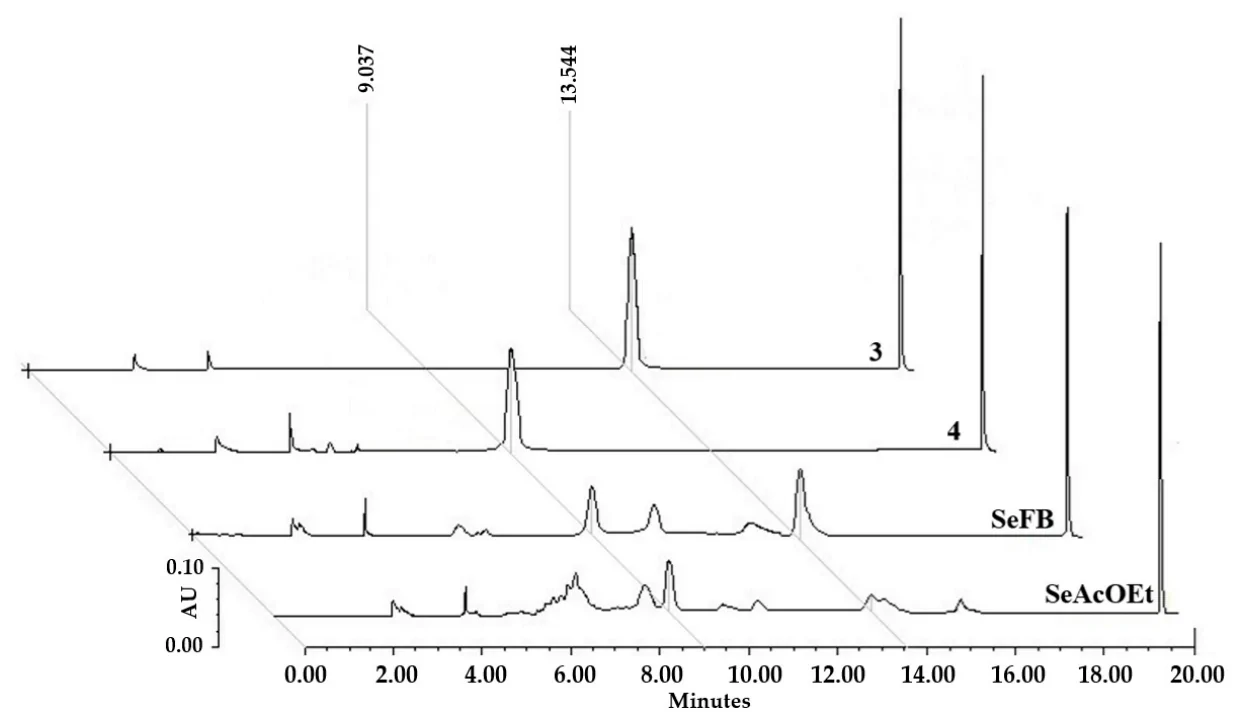
HPLC chromatogram of acetate of ethyl extract (SeAcOEt), fraction SeFB, and ursolic acid (UA, **3**) with a Rt = 13.54 min, 7α-hydroxysandaracopimaric acid (**4**) with a Rt = 9.04 min were identified.

According to the HPLC analysis (Figure 2) of the ethyl acetate extract (SeAcOE), the concentration of ursolic acids (**3**, Rt 13.5 min) and 7α-hydroxysandaracopimaric (**4**, Rt 9.0 min) was 0.039 and 0.045 mg/g, respectively. While in the SeFB fraction, these compounds are found at a concentration of 0.43 mg/g of **3** and 0.19 mg/g of **4**.

**Figure 3:**
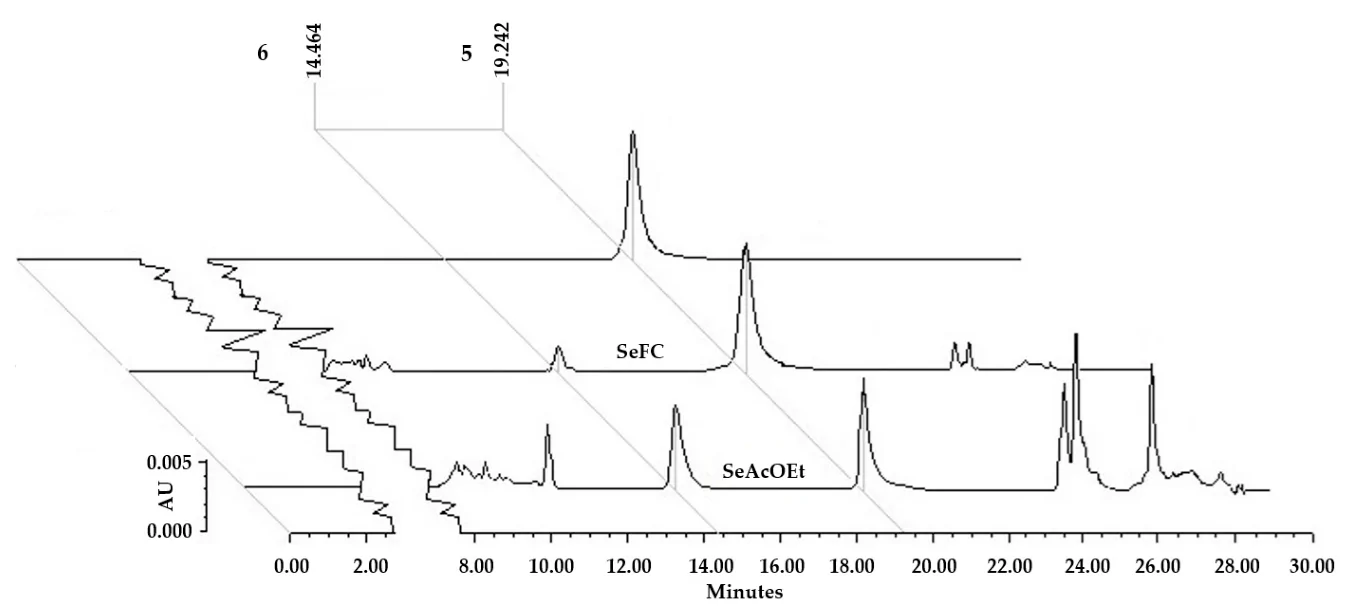
HPLC chromatogram of acetate of ethyl extract (SeAcOEt), fraction SeFC, and jaceosidin (**5**) with a Rt = 19.2 min and isosakuranetin-5-O-rutinoside (**6**) with a Rt = 14.46 min were identified.

When processing the SeFC fraction, the following two flavones were purified: jaceosidin **5** and isosakuranetin-5-O-rutinoside **6**, with retention times of 19.2 and 14.4 min, respectively (Figure 3).

**Figure 4:**
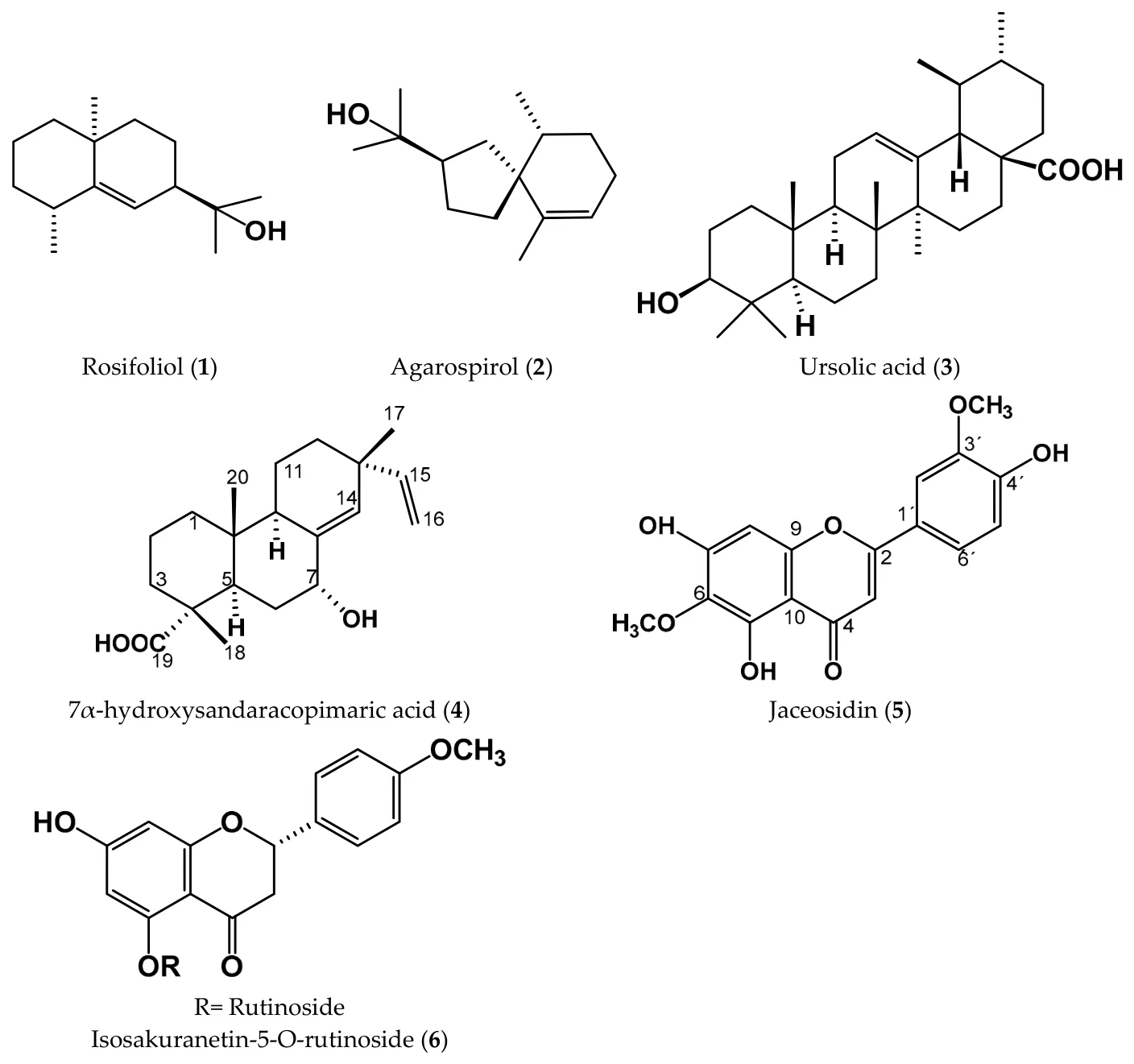
Chemical structures of isolated compounds from different fractions: SeFA (**1**/**2** terpenes mixture), SeFB (**3**,**4**), and SeFC (**5**,**6**), all of them from SeAcOEt and derivatives of *Salvia elegans*.

Compound (**5**) was isolated as a yellow powder with a melting point of 260–262 °C. In the UV light spectrum, the compound showed a λ_max_ 213.4, 254, 273 and 344 nm, characteristic of a flavone. The ^1^H NMR spectra of **5** showed the following three systems: aromatics A system [δ 6.71 (1H, s, H-8)]; signals other aromatic ABX system [δ 7.43 (1H, d, 1.9 Hz, H-2′), 6.9 (1H, d, 8.9 Hz, H-5′) and 7.45 (1H, dd, 1.95, 7.8 Hz, H-6′)] and double bond system in δ 6.85 (1H, s, H-3), which indicates a flavone nucleus called 6-hydroxiluteolin. Additionally, two proton signals of oxygenated base were observed in δ 3.73 and 3.91 that integrate for three protons and correlate in the HSQC with the carbon signal in δ 56.39 and 60.0 respectively, characteristic of two methoxyls. The methoxyl position was defined unambiguously to be at C-6 (δ 131.86) due to the long-range correlation (HMBC) between protons OCH_3_ (δ 3.73) and C-3′ (δ 149.81) with OCH_3_ (δ 3.91) respectively. Based on this information, the natural product was identified as jaceosidin. Based on direct comparison of spectroscopic data (see Table 2) described in the literature, this compound was identified as 4′,5,7-trihydroxy-3′,6-dimethoxyflavone or named jaceosidin (**5**, Figure 4) [21].

Compound (**6**, Figure 4) was isolated as an amorphous solid and identified as isosakuranetin-5-O-rutinoside. Structural identification of this flavanone was compared directly to a previously isolated sample and for HLPC (Figure 1 and Figure 4) [17].

### 2.2. Anti-Inflammatory Effect of Extract, Fractions and Compounds from Salvia elegans

**Figure 5:**
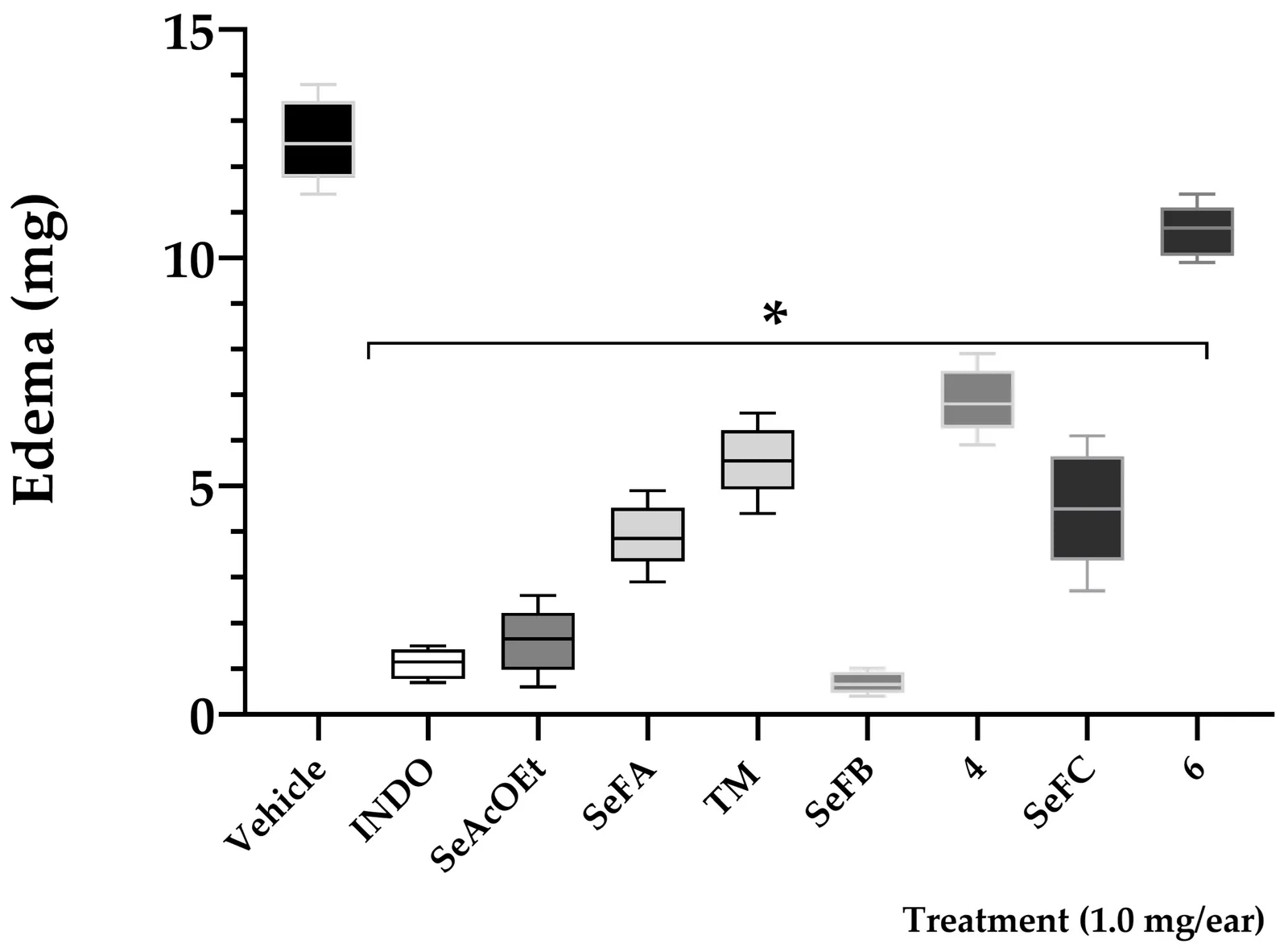

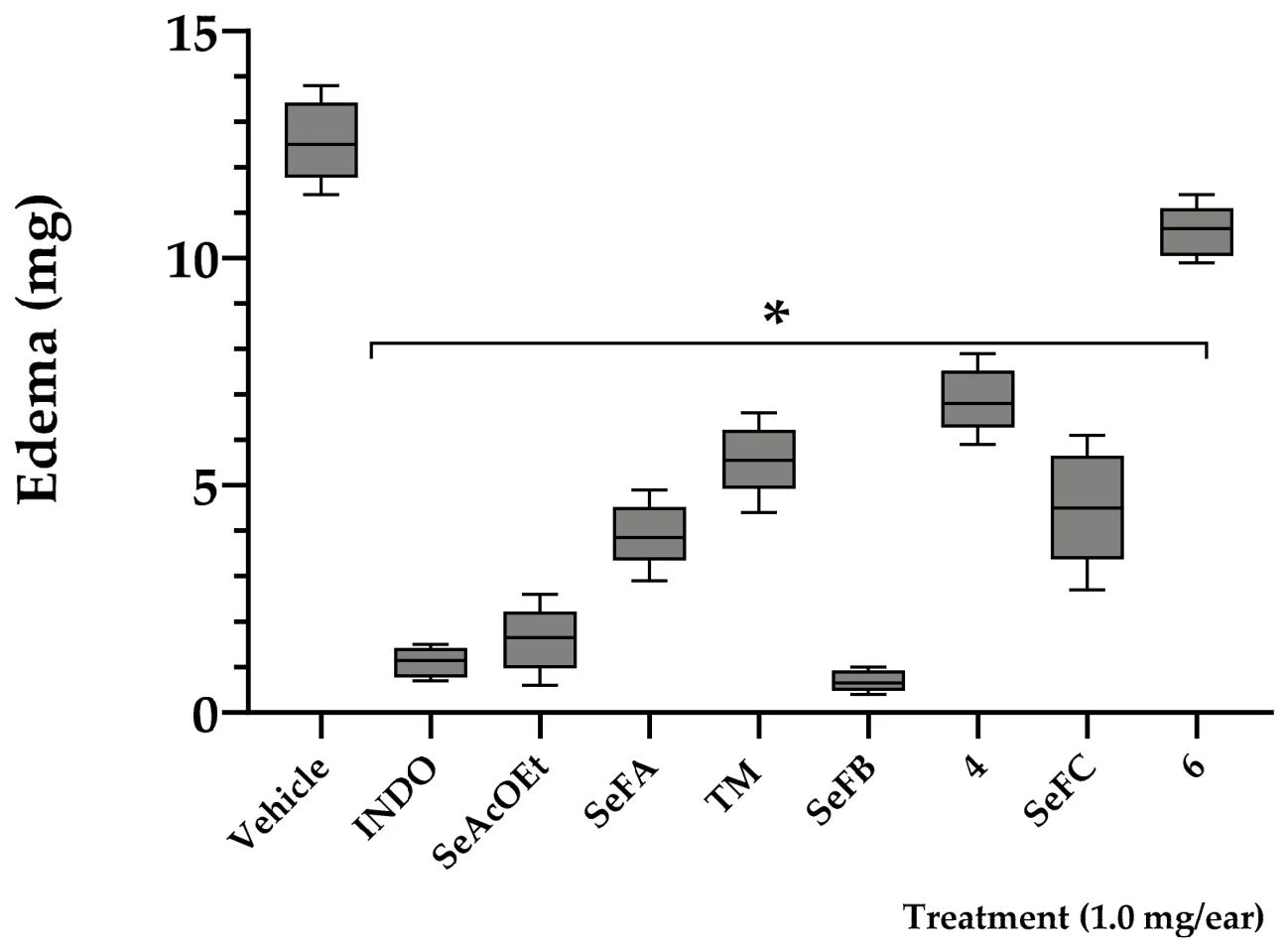
Effect of ethyl acetate extract (SeAcOEt), fractions and their compounds on mice auricular edema induced with a phorbol acetate ester (TPA). Least polar fraction: SeFA; terpenes mixture TM: Rosifoliol **1**, and Agarospirol **2**; medium polar fraction: SeFB; 7α-hydroxysandaracopimaric acid: **4**; polar fraction: SeFC; Isosakuranetin-5-O-rutinoside: **6**. Vehicle = vehicle group (acetone and TPA); indomethacin: INDO. ANOVA post hoc Dunnet * *p* < 0.05 in comparison with VEH (*n* = 6, ±ED).

The edema caused by TPA application in the mouse ear generated a differential of up to 12.6 ± 1.2 mg between the ear without damage and the inflamed ear. Treatment with topical administration of indomethacin (INDO) 1 mg/ear caused a decrease of about 91.2% in inflammation, a statistically significant decrease compared with TPA (1.1 ± 0.4 mg). Administration of 1 mg/ear of the *S. elegans* ethyl acetate extract (SeAcOEt) resulted in a decrease in edema (1.6 ± 1.0 mg), corresponding to an anti-inflammatory effect of 87.3%, which was significantly different from TPA data (Figure 5).

The fractionation of the SeAcOEt extract generated three fractions (Figure 5), where the SeFA corresponds to the least polar fraction, containing diterpenes and fatty acids. From the SeFA fraction (69.1%), the mixture of terpenes (TM) **1** and **2** was separated, which induced an inhibition of 55.7% (inflammatory edema of 5.5 ± 1.08 mg).

The SeFB fraction decreased the edema by 0.677 ± 2.1 mg, which represented a 97.4% inhibition of inflammation. It should be mentioned that the composition of this fraction mainly includes UA **3** and 7α-hydroxysandaracopimaric acid **4**, as observed in the chromatogram shown in Figure 2.

Fraction SeFC showed a moderate anti-inflammatory effect. It had the capacity to reduce edema up to 4.4 ± 1.7 mg, representing 65.6% inhibition of inflammation, and was identified in this fraction as jaceosidin, the major compound. Finally, the evaluation of the anti-inflammatory effect of isosakuranetin-5-O-rutinoside **6** was performed and showed no statistically significant difference with respect to the damage group (*p* > 0.05, VEH).

**Figure 6:**
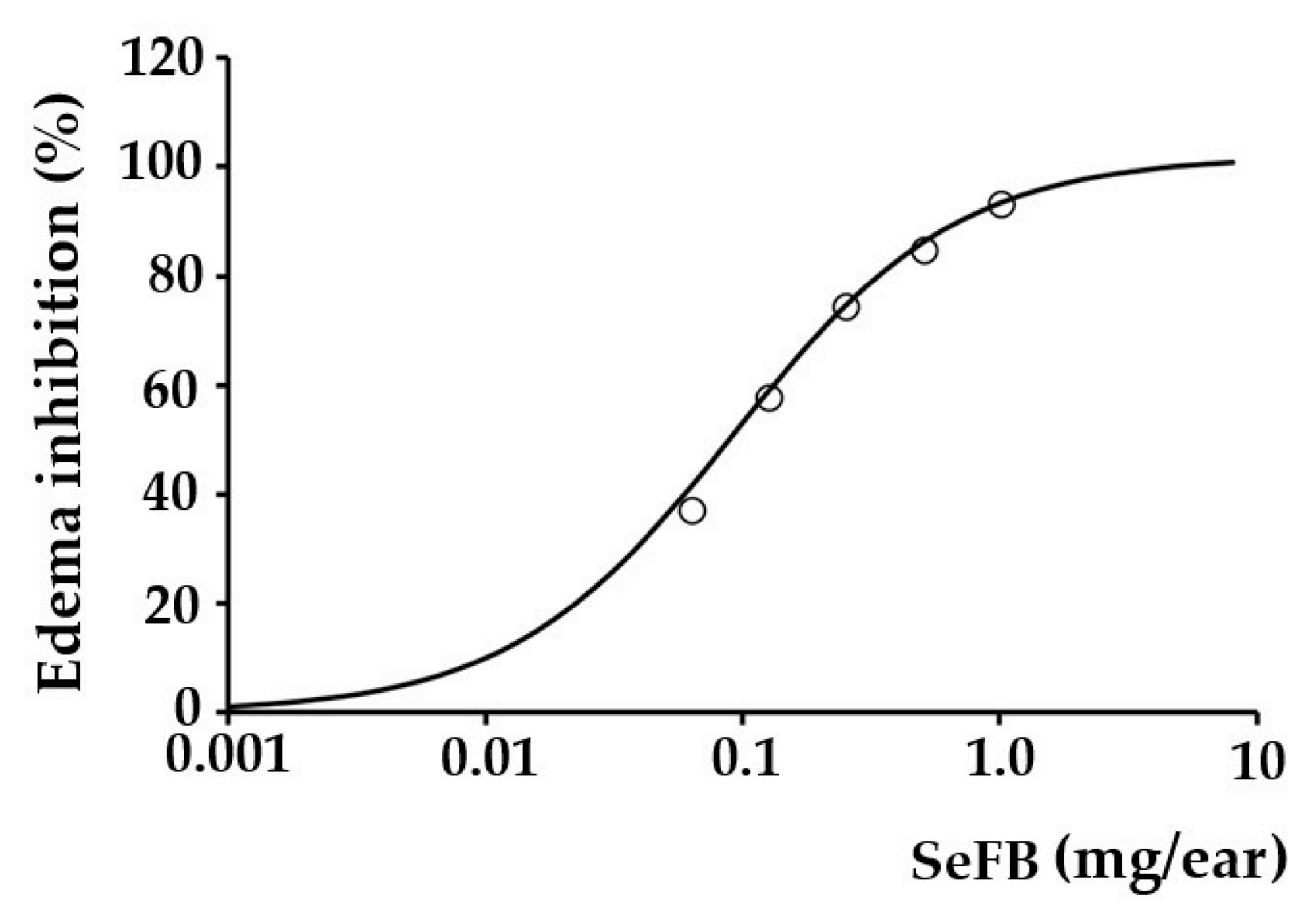
Dose–response curve of auricular edema produced by the topical application of phorbol ester acetate (TPA) and the SeFB fraction. In each point of the curve, *n* = 6.

**Figure 7:**
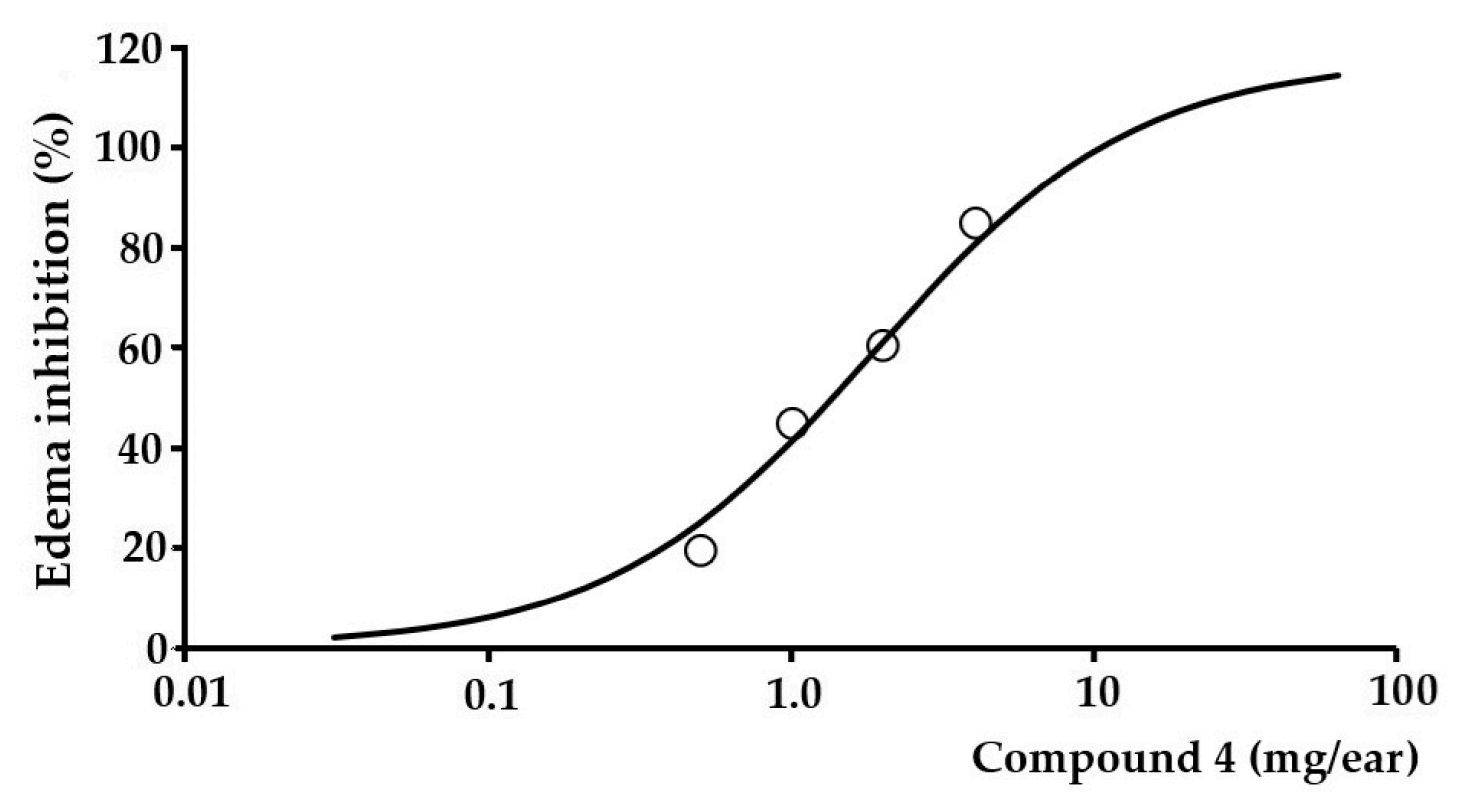
Dose–response curve of edema produced by the topical application of phorbol ester acetate (TPA) and 7α-hydroxysandaracopimaric acid (**4**). In each point of the curve, *n* = 6.

Because the SeFB fraction showed one of the most significant anti-inflammatory effects, a dose–effect curve was performed (Figure 6). In this dose–response behavior, the pharmacological constants were calculated as follows: E_max_ = 102% and ED_50_ = 0.092 mg/ear. It was also possible to establish that this fraction contained UA (**3**) and 7α-hydroxysandaracopimaric acid (**4**); then, a dose–response curve was performed with the new compound **4**, with E_max_ = 17% and ED_50_ = 1.087 mg/ear (Figure 7).

**Figure 8:**
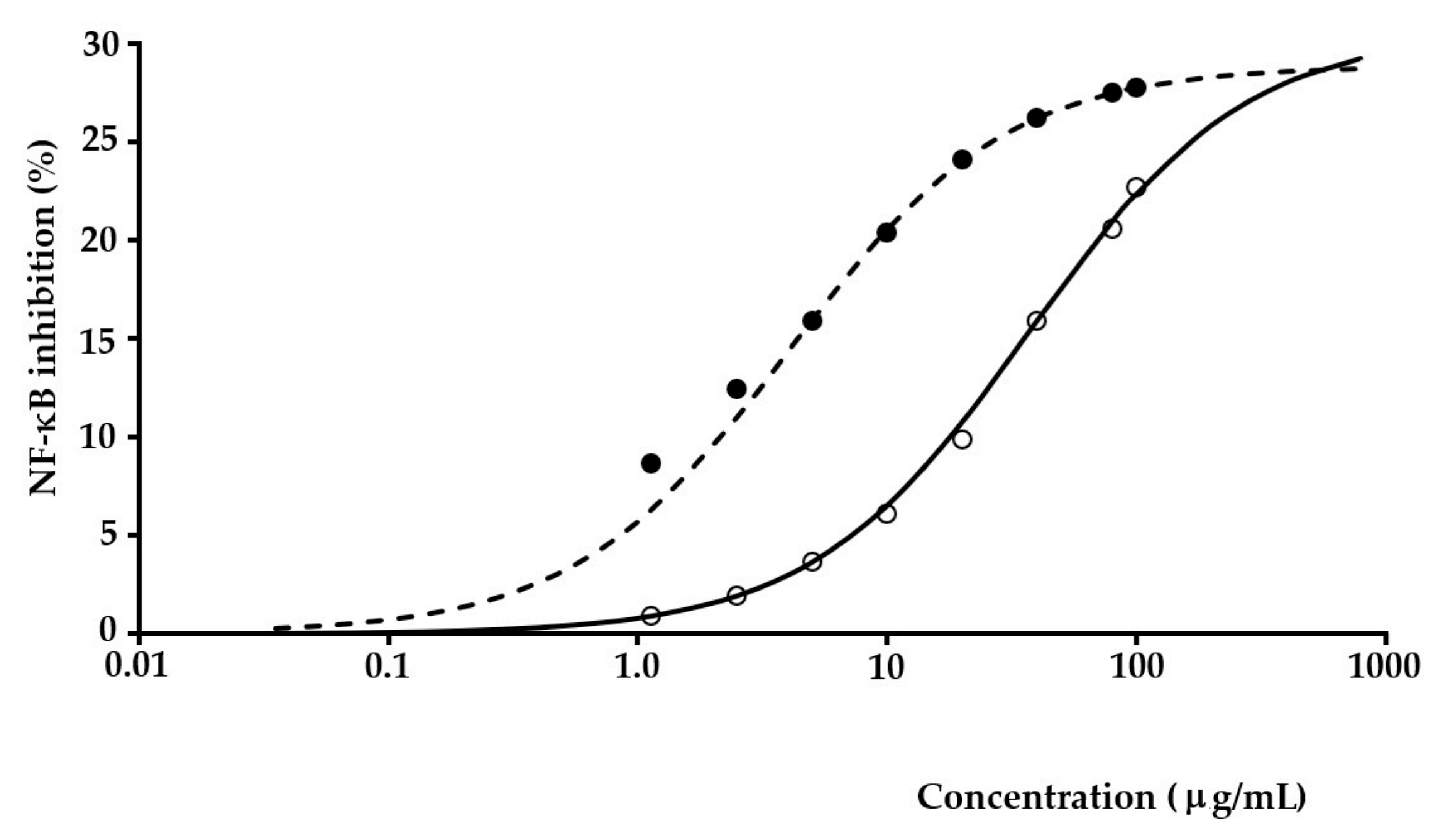
Concentration–response curve of the inhibition of NF-κB activation in RAW-Blue^TM^ cells stimulated with LPS. The number of repetitions was *n* = 4. Rosifoliol/agarospirol **1/2** TM (●) E_max_ = 28.9% and IC_50_ = 4.08 µg/mL, and 7α-hydroxysandaracopimaric acid (○) Emax = 36.6% and IC50 = 37.0 µg/mL.

Based on these results, a concentration–response curve in the RAW-Blue^TM^ cells in vitro assay was performed for **4** and **1**/**2** (TM). The data analysis indicates inhibition of NF-κB activation by both substances, with pharmacodynamic parameters of Emax = 28.9% and IC_50_ = 4.08 mg/mL for TM; meanwhile, for compound **4**, the values were Emax = 36.6% and IC_50_ = 37.0 mg/mL (Figure 8).

## 3. Discussion

The present study demonstrates that *S. elegans* Vahl. exhibits local anti-inflammatory activity, which could be attributable to a synergistic combination of terpenoid and flavonoid compounds. The bio-guided investigation using the TPA-induced mouse ear edema model allowed the isolation of the following six main metabolites: mixture 1/2, 3, 4, 5, and 6. The identification of these compounds confirms that this species shares the pharmacological potential observed in other members of the Salvia genus, such as *S. connivens* and *S. plebeia*, in which terpenoid and flavonoid extracts have been shown to inhibit pro-inflammatory mediators such as COX-2 and to inhibit NF-κB signaling [22,23].

For the identification of the active compounds in *S. elegans*, the TPA-induced mouse ear edema model was employed; this compound induces a marked inflammatory response in the skin characterized by neutrophil infiltration [24]. TPA activates phospholipase A_2_ (PLA_2_), promoting the release of arachidonic acid (AA) and consequently stimulating prostaglandin synthesis [25]. The biochemical cascade that converts AA into prostaglandins is triggered by diverse stimuli, including mechanical stress, heat, chemical injury, bacterial products, and pro-inflammatory cytokines [26]. Activation of this eicosanoid pathway involves the NF-κB signaling pathway, which depends on COX-2 expression induced by TPA [27,28]. INDO, a known COX-2 inhibitor, was used as a positive control; its predictable anti-inflammatory effect confirms and validates the reliability of the experimental model [29]. This study shows that SeFB has a greater effect than INDO, which may be because this natural product contains COX-2-inhibiting compounds such as ursolic acid (**3**), a well-documented triterpene previously reported to show significant anti-inflammatory activity in the TPA-induced ear edema model, with an ED_50_ value of 0.02 mg/ear [22], and also mediated the direct inhibition of COX-2 and the nuclear translocation of NF-κB [28,29]. Therefore, SeFB could act through multiple anti-inflammatory mechanisms via its secondary metabolites, producing a greater effect than INDO. This suggests a pharmacological interaction among the compounds in the fraction. Consequently, this superior effect could be investigated using isobologram assays with isolated compounds. This approach would help determine whether the components in SeFB exhibit synergy.

**Figure 9:**
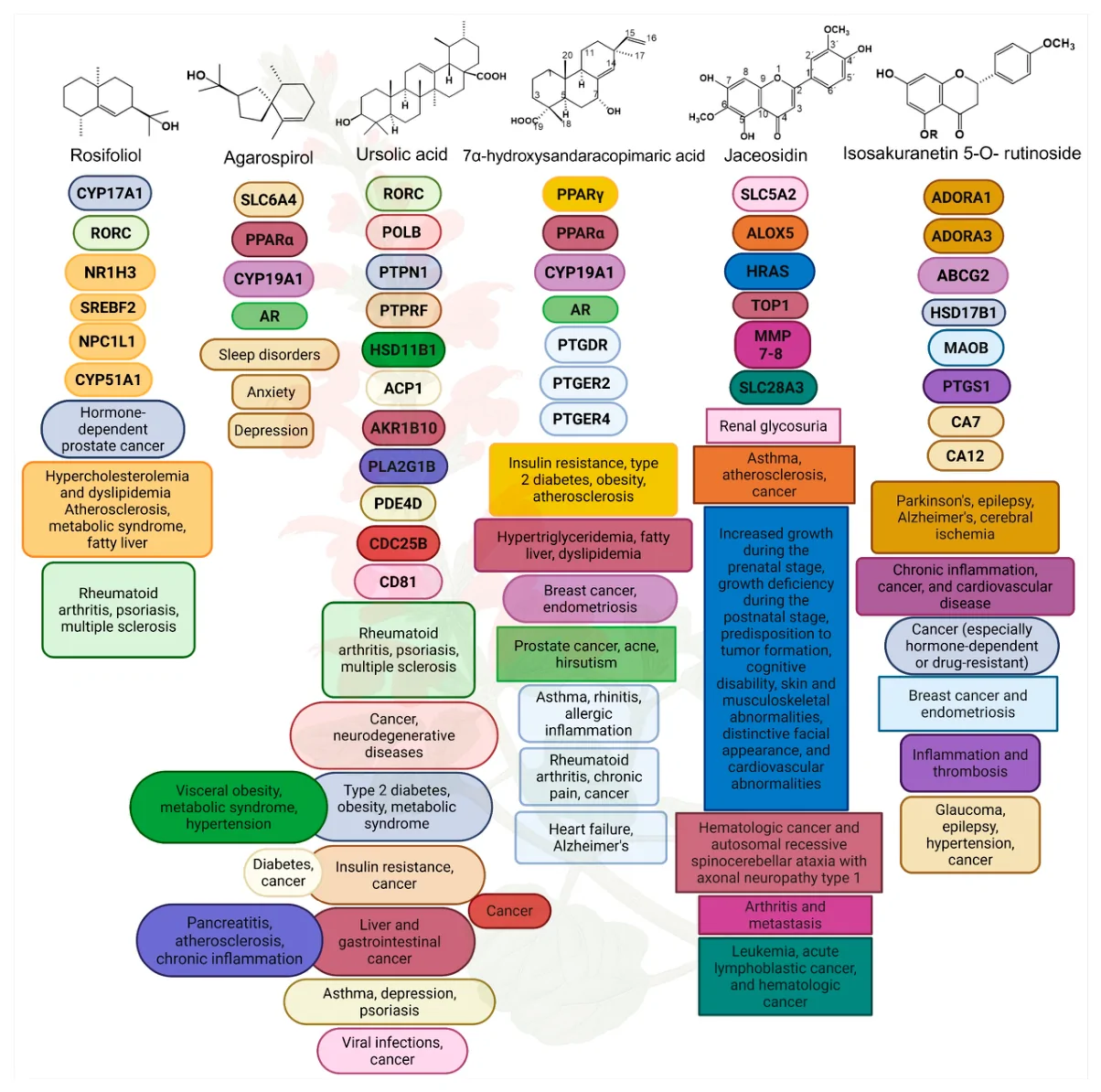
Prediction of drug targets is likely to interact with ligands isolated from *Salvia elegans* extracts using SwissTargetPrediction. PPARG Gene—peroxisome proliferator activated receptor gamma; CYP19A1 gene—cytochrome P450 family 19 subfamily A Member 1; PPARA gene—peroxisome proliferator activated receptor alpha; PTGDR gene—prostaglandin D2 receptor; MME gene—membrane metalloendopeptidase; PTGER2 gene—prostaglandin E receptor 2; AR gene—androgen receptor; PTGER4 gene—prostaglandin E receptor 4; CYP19A1 gene—cytochrome P450 family 19 subfamily A member 1; SLC6A4 gene—solute carrier family 6 member 4; NR1H3 gene—nuclear receptor subfamily 1 group H member 3; CYP17A1 gene—cytochrome P450 family 17 subfamily A member 1; RORC gene—RAR-related orphan receptor C; SREBF2 gene—sterol regulatory element binding transcription factor 2; NPC1L1 gene—NPC1-like intracellular cholesterol transporter 1; ADORA1 gene—adenosine A1 receptor; ADORA3 gene—adenosine A3 receptor; ABCG2 gene—ATP binding cassette subfamily G member 2 (JR blood); HSD17B1 gene—hydroxysteroid 17-beta dehydrogenase 1; MAOB gene—monoamine oxidase B; PTGS1 gene—prostaglandin-endoperoxide synthase 1; CA7 gene—carbonic anhydrase 7; CA12 gene—carbonic anhydrase 12; SLC5A2 gene—solute carrier family 5 member 2; ALOX5 gene—arachidonate 5-lipoxygenase; HRAS gene—HRas proto-oncogene, GTPase; TOP1 gene—DNA topoisomerase I; MMP7 gene—matrix metallopeptidase 7; MMP8 gene—matrix metallopeptidase 8; SLC28A3 gene—solute carrier family 28 member 3; RORC gene—RAR-related orphan receptor C; POLB gene—DNA polymerase beta; PTPN1 gene—protein tyrosine phosphatase non-receptor type 1; PTPRF gene—protein tyrosine phosphatase receptor type F; PTPN2 gene—protein tyrosine phosphatase non-receptor type 2; HSD11B1 gene—hydroxysteroid 11-beta dehydrogenase 1; ACP1 gene—acid phosphatase 1; AKR1B10 Gene—aldo-keto reductase family 1 member B10; PLA2G1B gene—phospholipase A2 group IB; CDC25B gene—cell division cycle 25B.

From this same fraction, 7α-hydroxysandaracopimaric acid was isolated for the first time from this species, and its anti-inflammatory activity was evaluated. The results demonstrated that this new compound effectively reduced ear edema. Furthermore, previous studies have described related pimarane diterpenes, such as pimaradienoic acid, an isomer of pimaric acid, which were shown to inhibit carrageenan-induced leukocyte and neutrophil recruitment in the peritoneal cavity, as well as to reduce carrageenan-induced paw edema [30]. These findings suggest that the pimarane structural framework plays a crucial role in the anti-inflammatory activity of this class of compounds. The prediction analysis with 7α-hydroxysandaracopimaric acid, isolated for the first time from *S. elegans*, exhibited anti-inflammatory activity, possibly mediated through the modulation of nuclear receptors of the PPAR type (α and γ). According to pharmacological predictions performed using SwissTargetPrediction, this diterpene interacts with key genes and proteins involved in lipid homeostasis and immunometabolic regulation, such as PPARG, PPARA, and MME, as well as prostaglandin receptors PTGER2, PTGER4, and PTGDR (Figure 9) [31]. These interactions suggest that 7α-hydroxysandaracopimaric acid may exert a pleiotropic effect, modulating both acute inflammation and chronic processes associated with metabolic and cardiovascular disorders. Activation of PPARγ is associated with the suppression of SEAP activation for NF-κB signaling and COX-2, which is consistent with the experimental model of TPA-induced edema inhibition. The presence of ursolic acid (**3**) reinforces the anti-inflammatory profile of SeFB and the SeAcOEt extract of *S. elegans* and contributes significantly (≈59%) to the total observed effect. The combined action of this compound with 7α-hydroxysandaracopimaric acid (**4**) suggests a biochemical synergy between these terpenes.

Jaceosidin (**5**), isolated from SeFC, also reported in this work for the first time in *S. elegans*, was not tested here for anti-inflammatory effect, due to this substance has been evaluated in biological models of inflammation, is recognized for its ability to suppress the expression of COX-2 and the production of TNF-α and IL-1β, as well as to inhibit the activation of NF-κB signaling [32,33,34].

In contrast, the isosakuranetin-5-O-rutinoside (**6**), present in the same fraction, showed no local anti-inflammatory effect at doses of 1 mg/ear, which may be attributed to structural differences affecting its interaction with enzymes and receptors involved in the inflammatory response, such as the absence of a hydroxyl group at the C-7 position [35], contrary to what is reported for its isomer sakuranetin, which did have the anti-inflammatory effect, due to diminishing the neutrophil recruitment to the peritoneal cavity of mice pretreated prior with carrageenan. In addition, it was mentioned that the activity was due to the presence of hydroxyl group at C-7 and methoxyl group at C-5 at sakuranetin [36] and then isosakuranetin-5-O-rutinoside isolated from *S. elegans* [10] has not these chemical characteristics.

The SeFA, from which the diterpenes rosifoliol (**1**) and agarospirol (**2**) could be isolated—the first one has reports of anti-inflammatory and cytotoxic activity; rosifoliol is a component of *Helichrysum microphyllum*, which has antioxidant activity in vitro [37].

From a molecular perspective, prediction analyses suggest that several of the identified metabolites act on relevant pharmacological targets. For instance, agarospirol and rosifoliol showed affinity for CYP19A1 (aromatase) and AR (androgen receptor), implying a potential modulatory role in the synthesis of sex steroids and, therefore, possible therapeutic applications in hormone-dependent diseases such as breast cancer, endometriosis, or benign prostatic hyperplasia [38,39].

In the case of rosifoliol, its potential interaction with NR1H3 (liver X receptor alpha, LXRα) and with genes such as SREBF2 and NPC1L1 suggests involvement in the regulation of cholesterol metabolism and lipid homeostasis [40,41]. Since activation of LXRα induces the transcription of genes involved in cholesterol efflux and the suppression of NF-κB-dependent inflammatory processes, rosifoliol could indirectly contribute to the systemic anti-inflammatory activity of the extract, in addition to offering potential benefits in pathologies such as atherosclerosis and dyslipidemia (Figure 9) [42].

Based on the above findings, the anti-inflammatory effect of the SeAcOEt extract is primarily attributed to the presence of ursolic acid **3**, 7α-hydroxysandaracopimaric acid **4**, jaceosidin **5**, and the rosifoliol/agarospirol **1**/**2**, TM. The concentrations of these compounds in the extract were determined to be 0.039, 0.045, 0.0097, and 0.019 mg per mg of extract, respectively. The IC_50_ values for these compounds in the TPA-induced mouse ear inflammation assay were 0.02, 1.087, and 0.0033 mg, respectively [35,43].

The TM exhibited an IC_50_ value of 4 μg/mL, although this was determined in a different biological model—specifically, the inhibition of the activation of NF-κB and AP-1 in RAW-Blue^TM^ cells cultures. Similarly, the IC_50_ value reported for jaceosidin was obtained in a pharmacological model distinct from TPA-induced inflammation; however, it provides a useful approximation of the expected anti-inflammatory potency of this compound.

If the extract’s effect results solely from a summative pharmacological interaction among its components, the relative contribution of each compound can be estimated as follows: 59.1% for **3**, 2.8% for **4**, 2.7% for **5**, and 5.0% for the **1/2** TM. The total calculated summative effect of 90.0% closely approximates the 87.3% inhibition observed for the SeAcOEt extract. Similarly, since the concentrations of the active compounds in fraction SeFB ursolic acid and 7α-hydroxysandaracopimaric acid were quantified at 0.43 and 0.19 mg per mg of fraction, respectively, their individual contributions correspond to 81.7% and 10.8% inhibition. The combined inhibitory effect of 92.6% aligns closely with the experimentally determined 98% inhibition for the SeFB fraction, further supporting the consistency and reliability of the calculated values.

The results obtained through SwissTargetPrediction (Figure 9) reinforce the idea that the metabolites present in *S. elegans* do not act independently but rather as part of a network of interactions with nuclear receptors, lipid metabolism enzymes, and G protein-coupled receptors involved in inflammation (PTGER2, PTGER4, and PTGDR) [44]. This integrative perspective suggests that the plant may simultaneously modulate metabolic, endocrine, and immunological pathways, conferring a multifunctional therapeutic profile that is particularly relevant in the context of chronic and metabolic inflammatory diseases.

Taken together, the experimental and computational findings support that the SeAcOEt of *S. elegans* could be act as anti-inflammatory probably and according to prediction analysis of the COX-2/NF-κB-dependent eicosanoid cascade and the activation of PPAR nuclear receptors. The isolated compounds—particularly **4**, **3**, **5**, and the TM **1/2**—could be act complementarily by regulating both local pro-inflammatory mechanisms and systemic processes related to metabolic and hormonal homeostasis [43,45].

These results open a new perspective for pharmacological research on *S. elegans*, highlighting its potential as a source of natural anti-inflammatory agents with possible applications in metabolic, cardiovascular, neurodegenerative, and hormone-dependent oncological diseases [20].

However, several limitations warrant consideration. The study was conducted primarily in an acute inflammation model (TPA-induced edema), which, although validated, may not fully represent chronic or systemic inflammatory conditions. Additionally, the target predictions, while informative, require experimental validation through receptor-binding assays, gene expression analyses, and refined molecular docking. The possibility that other molecular mechanisms—such as COX-2 inhibition and cytokine modulation, among others—contribute to the activity of the evaluated compounds cannot be ruled out; further experiments are required to investigate this. The lack of pharmacokinetic and toxicity data for the isolated compounds also limits the immediate translational applicability of the findings.

## 4. Materials and Methods

### 4.1. General Experimental Procedures

Melting points were obtained on a Thermo Scientific IA9000 series (Waltham, MA, USA) melting point apparatus and were uncorrected. All NMR spectra were recorded on Varian INOVA-400 (Varian, Palo Alto, CA, USA) at 400 MHz for ^1^H NMR, NOESY, ^1^H-^1^H COSY, HSQC and HMBC, and 100 MHz for ^13^C NMR and DEPT in CDCl_3_. Chemical shifts are reported in ppm relative to TMS.

HPLC analysis was performed on a Waters 2695 (Milford, MA, USA) separation module system equipped with a Waters 2996 photodiode array detector and Empower Chromatographic Manager version 1 software (Mildford, MA, USA). GC-MS equipment used for the identification of the compounds was an Agilent Technology 6890 plus Gas Chromatograph (Santa Clara, CA, USA), coupled to a 5973N mass spectrometer with a simple quadrupole analyzer, with the Electron Impact (IE) ionization mode at 70 eV [38]. The equipment has an automatic injection system. Compounds in a mass range of 30 to 500 Dalton can be optimally detected. Volatile and non-thermolabile organic compounds were separated on a HP 5MS capillary column (25 m long, 0.2 mm i.d., with 0.3 µm film thickness). Oven temperature was set at 40 °C for 2 min and then programmed from 40–260 °C at 10 °C/min and maintained for 20 min at 260 °C. Mass detector conditions were as follows: interphase temperature 200 °C and mass acquisition range, 20–550. Injector and detector temperatures were set at 250 and 280 °C, respectively. Splitless injection mode was carried out with 1 µL of each fraction (3 mg/mL solution). The carrier gas was helium at a flow rate of 1 mL/min.

### 4.2. Plant Material

The aerial parts of *Salvia elegans* (6 kg) were collected in Ozumba, the state of México, 19°02′20.58″ north latitude and 98°47′42.38″ longitude west. The voucher specimen of this material (2056) was deposited in the Botanical Garden and Museum of Traditional Medicine and Herbal Medicine of INAH Morelos, identified by the taxonomists Margarita Aviles and Macrina Fuentes Mata.

### 4.3. Extract Preparation

The fresh material was dried in the shade, in a ventilated place and at a temperature of 25 °C. Subsequently, the dry leaves (3 kg) were ground in a pulvex mill and placed in a glass container for extraction with ethyl acetate 15 L (Merck, Darmstadt, Germany), allowing to rest for 24 h at room temperature. This process was carried out in triplicate. The ethyl acetate extract was filtered and concentrated in a rotary evaporator Heidolph Laborota G3 (Schwabach, Germany), under reduced pressure at 45–50 °C until completely dry, which was frozen with the help of a lyophilizer Labconco (Saint Louis, MO, USA) to give 330 g of the SeAcOE extract.

### 4.4. Chromatographic Fractionation of SeAcOE Extract

Ethyl acetate extract (200.3 g) was adsorbed on silica gel and applied to a glass column (10 × 50 cm) packed with normal phase silica gel (400.5 g, 70-230 mesh, Merck). An n-hexane/ethyl acetate/methanol gradient was used as the mobile phase for column elution. A total of 500 mL aliquots were collected and concentrated using a rotary evaporator under reduced pressure, yielding 61 fractions. These were grouped into 17 fractions (SeF1–SeF17) based on compound similarity. Thin-layer chromatography analysis of the fractions allowed for their regrouping into three subfractions (SeFA, SeFB and SeFC) for evaluation in the TPA-induced edema model.

### 4.5. Chromatographic Fractionation of SeFA, SeFB and SeFC Fractions

One of the active fractions (SeFA) was analyzed by gas masses (GC-MS) and rosifoliol **1** and agarospirol (**2**) were identified [19].

SeFB fraction (5g) was adsorbed silica gel and applied to a column of silica gel (150 g, 70-230 mesh, Merck, Darmstadt, Germany). A gradient of *n*-hexane/ethyl acetate/methanol was used to elute the column, collecting 24 fractions of 200 mL each. Fractions were grouped according to their similarity in TLC in 20 fractions (SeR1-SeR20).

Fractions SeR5 to SeR7 showed a single spot on TLC. HPLC and mass analysis allowed the identification of ursolic acid (3). The SeR11-SeR15 fractions (626.6 mg) were pooled and purified on a reversed-phase column chromatography (10 g, RP-18, 40–63 µm, Merck, Darmstadt, Germany) with a water/acetonitrile mobile phase in a gradient system starting with 70:30 and increasing polarity by 10%. A total of 10 mL samples were collected, yielding 41 fractions. Fractions 33–36 (SeFB) showed a single spot on TLC and were identified by one- and two-dimensional NMR analysis as a diterpene called 7α-hydroxysandaracopimaric acid (**4**).

HPLC analysis was performed with a column LiChrospher^®^ (Rp18, 20 × 5 μm, 50 μm) (Merck KGaA, Darmstadt, Germany), at a flow rate of 1.20 mL/min, and a gradient system water/acetonitrile (A: B), having the following proportions A: B; 100:0 (0–1 min); 60:40 (2–3 min); 10:90 (4–10 min); 0:100 (11–17 min); and 100:0 (18–20 min). Total running time of samples was 20 min, with 10 μL injection.

The detection wavelength was scanned at 190–220 nm. This method was used for ursolic acid (**3**) and 7α-hydroxypimaric acid (**4**). The retention times (Rt) to compounds **3** and **4** were 13.54 and 9.03 min, respectively.

### 4.6. Isolation of Jaceosidin and HPLC Analysis

SeFC fraction (8 g) was separated by using a chromatographic column with silica gel (200 g, 70-230 mesh, Merck, Darmstadt, Germany), eluting with a solvent mixture with increasing polarity with hexane/ethyl acetate/methanol, collecting 14 fractions of 200 mL each. Fractions 7 and 8 were combined and recrystallized for obtaining solid yellow, which was elucidated by analysis of ^1^H and ^13^C NMR as jaceosidin (**5**, 30 mg), a known flavonoid. In fraction 10, the flavonoid known as Isosakuranetin-5-O-rutinoside (**6**, 100 mg) was identified. Spectroscopic data were compared with the literature [17].

HPLC analysis was performed with a column SUPERCOSIL^TM^ LC-F de 25 cm × 4.5 mm, 5 μm (Merck KGaA, Darmstadt, Germany), at a flow rate of 0.90 mL/min, and a gradient system water-TFA 0.5% and acetonitrile (A: B) having the following proportions: A: B 100:0 (0–1 min); 95:5 (2–3 min); 70:30 (4–20 min); 50:50 (21–23 min); 20:80 (24–25 min); 0:100 (26–27 min); and 100:0 (28–30 min). The total running time of samples was 30 min, with 10 μL injection. The detection wavelength was scanned at 345 nm. The retention times (Rts) of compounds **5** and **6** were 19.2 and 14.6 min, respectively.

### 4.7. Animals

All procedures were conducted in accordance with Official Mexican Norm (NOM-062-ZOO-1999) [46], regarding technical specifications for the production, care, and use of laboratory animals, and the Guide for the Care and Use of Laboratory Animals and the international ethical guidelines for the care and use of laboratory animals. ICR healthy male albino mice weighing between 25 and 30 g (Harlan, Mexico City, Mexico) were used for this assay. All animals were housed eight per cage and maintained under laboratory conditions at 25 °C, with a normal 12 h:12 h light/dark schedule (lights on at 7:00 a.m.) and free access to water and food (pellets, Envigo rodent lab diet, Indianapolis, IN, USA). The mice were allowed 3 weeks to adapt to the laboratory environment prior to the trial period. Experiments were carried out between 8:00 a.m. and 12:00 p.m. This research study was approved by the ethical committee of the Mexican Social Security Institute (R-2023-1702-010). Minimum number of animals and minimum duration required to obtain consistent data were employed.

### 4.8. Anti-Inflammatory Activity Assay in Ear Edema Induced with TPA

The isolated compounds and fractions were evaluated in the anti-inflammatory assay, which was carried out following the method described by Payá [47]. A total of 60 animals were randomly distributed into 10 groups of 6 mice each; they were separated into cages for technician personnel who did not do the test. In our experience, this sample size allows the reproducibility of the assay and adequate statistical management. Each group received 12-O-Tetradecanoylphorbol-13-acetate (TPA −2.5 μg) (Sigma-Aldrich, Darmstadt, Germany) dissolved in acetone (20 μL), which was applied on the internal and external surface on the right ear to cause edema. Doses of 1 mg/ear of each treatment INDO, SeAcOEt, SeFA, SeFB, SeFC, 7α-hydroxypimaric (**4**) acid, isosakuranetin-5-O-rutinoside (**6**), and mixture of agarospirol/rosifoliol (**1**/**2**) were applied to the ear of each mouse. All treatments were dissolved in acetone and applied topically, 10 min before administration of TPA. Mice were sacrificed by overdoses of sodium pentobarbital and after cervical dislocation, in the 6 h at the start of the assay. Subsequently circular sections of 6 mm diameter were obtained from both ears: one with inflammation with TPA (t) and without TPA (nt). With the weights of each tissue section, we calculated the percentage inhibition of inflammation. Percentage of inhibition was obtained using expression below:

Inhibition % = (Δw control − Δw treatment/Δw) × (100),

where

Δw = wt − wnt

wt is the weight of the section of the treated ear;

wnt is the weight of the section of the non-treated ear.

### 4.9. Anti-NF-κB Activity Assay in RAW-Blue^TM^ Macrophages Stimulated with LPS

The evaluation of the anti-inflammatory activity of *Salvia elegans* compounds was carried out using a cell line of macrophages (RAW-Blue^TM^ cells, InvivoGen, Catalog code: Raw-Sp. San Diego, CA, USA). These cells have a reporter gene that expresses an alkaline phosphatase (SEAP). The enzyme is under the control of an inducible promoter, by the transcription factors NF-κB and AP-1. In addition, the SEAP that is secreted into the culture medium catalyzes the enzymatic transformation of the substrate, which allows the reaction to be followed by using a colorimeter.

The cells were grown and maintained in DMEM, according to the manufacturer’s instructions (Dulbecco’s modified Eagle’s Medium) (Waltham, MA, USA) at 37 °C and 5% of atmosphere CO_2_. The assay was carried out in the following manner: the cells were inoculated in 24 well plates with 1 × 10^5^ cells/well and were incubated for six hours; subsequently, the macrophages were stimulated with 20 ng/mL of lipopolysaccharide (LPS) (Sigma-Aldrich, Darmstadt, Germany) and, at the same time, increasing amounts (1.25, 2.50, 5.00, 10.00, 20.00, 40.00 and 80.00 μg/mL) of the mixture of terpenes (rosifoliol/agarospirol **1/2**, TM) or 7α-hydroxysandaracopimaric acid (**4**) were administered, and the cells were incubated for 12 h. At the end of the incubation period, 50 μL of culture medium was collected, on which the alkaline phosphatase activity was determined using the QUANTI-Blue reagent (InvivoGen) (San Diego, CA, USA) to quantify the enzymatic activity. There were three independent replicates (*n* = 4). Experiments were repeated on different days and at different passages to account for biological variation.

### 4.10. SwissTargetPrediction. Prediction of Different Pharmacologic Targets

The chemical structures of **1**, **2**, **3**, **4**, **5**, and **6** were obtained from previously reported structural data and converted into canonical SMILES format for use with PUBCHEM (pubchem.ncbi.nlm.nih.gov). Subsequently, target prediction was performed using the SwissTargetPrediction web server (https://www.swisstargetprediction.ch/ accessed on 25 January 2026), with Homo sapiens selected as the reference organism. Predicted targets were ranked by probability score (Appendix A).

### 4.11. Statistical Analysis

Data from TPA and cell culture assays were analyzed by ANOVA, with a post-test Dunnett in comparison with vehicle group; any experimental unit was excluded, with statistical differences established at * *p* < 0.05, by using the SPSS 20.0 program.

## 5. Conclusions

The present study provides the first comprehensive pharmacological and phytochemical characterization of the ethyl acetate extract of *Salvia elegans*, integrating chemical profiling with complementary in vivo, in vitro, and computational approaches. The results demonstrate that the extract exhibits significant local anti-inflammatory activity, while bioassay-guided fractionation identified SeFB as the most active fraction, enriched in ursolic acid and the newly identified diterpene 7α-hydroxysandaracopimaric acid.

Detailed spectroscopic analysis established, to the best of our knowledge, the first report of 7α-hydroxysandaracopimaric acid in *S. elegans*, thereby expanding the phytochemical knowledge of this medicinal species. Pharmacological evaluation further demonstrated that the terpene mixture (rosifoliol/agarospirol, TM) and 7α-hydroxysandaracopimaric acid reduced NF-κB/AP-1-dependent SEAP reporter activity in LPS-stimulated RAW-Blue™ macrophages, supporting their contribution to the anti-inflammatory activity of the extract. However, the markedly higher activity observed for SeFB than for compound **4** alone suggests that the pharmacological effect of the extract is more likely explained by the combined contribution of multiple metabolites rather than by a single active constituent.

Computational target prediction suggested that several of these metabolites may interact with molecular targets involved in inflammatory signaling, including prostaglandin receptors, nuclear receptors, and lipid metabolism-related proteins. Nevertheless, these predictions should be regarded as hypothesis-generating, and they require experimental validation before mechanistic conclusions can be drawn.

Overall, this study provides pharmacological evidence supporting the traditional use of *S. elegans* for inflammatory conditions and establishes a solid foundation for future investigations aimed at defining the individual and combined contributions of its major metabolites, validating their molecular targets, and elucidating the mechanisms underlying the anti-inflammatory activity of this medicinal species.

## Figures and Tables

**Table 1:** NMR spectroscopy data (^1^H 400 MHz, ^13^C 100 MHz, in CD_3_OD) of 7α-hydroxysandaracopimaric acid (**4**).

Position	δ^13^_C_	Type	δ ^1^_H_ (*J* in Hz)
1α β	39.48	CH_2_	1.81–1.75 (m) 1.22 (dd, 12.0, 5.5)
2	19.42	CH_2_	1.67–1.55 (m)
3α β	38.0	CH_2_	1.89 (td, 13.6, 7.4) 1.58 (m)
4	47.9	C	
5	43.2	CH	2.42 (dd, 2.5, 13.1)
6α Β	33.1	CH_2_	1.68, ddd (3.0, 9.8, 13.0) 1.42 (q, 2.4, 3.1)
* 7	73.7	CH	4.10, dd (2.6, 2.7)
8	139.7	C	
9	47.2	CH	2.22, ddd (1.4, 8.4, 8.4)
10	39.0	C	
11α β	19.2	CH_2_	1.58 (m) 1.61 (m)
12 α β	35.3	CH_2_	1.46 (d, 3.2) 1.42 (m)
13	38.4	C	
14	135.0	CH	5.51 (d, br, 1.41)
15	149.2	CH	5.80 (dd, 10.6, 17.5)
16α β	111.3	CH_2_	4.91, dd (1.4, 10.6) 4.99, dd (1.4, 17.5)
17	26.2	CH_3_	1.05, s
18	182.3	C	
19	17.5	CH_3_	1.17, s
20	15.1	CH_3_	0.85, s

* The α-orientation of the hydroxyl group at C-7 was assigned from the NOESY correlations observed for H-7 (δH 4.10) with H-20 (δH 0.85), H-14 (δH 5.51), H-6a (δH 1.68), and H-6b (δH 1.42). The spectroscopic data were consistent with those previously reported for 7α-hydroxysandaracopimaric acid [20].

**Table 2:** NMR spectroscopy data (^1^H 400 MHz, ^13^C 100 MHz, in DMSO-*d*_6_) of jaceosidin (**5**).

Position	δ ^13^C	δ ^1^H (*J* in Hz)
2	164.23	
3	102.71	6.85 (s)
4	182.09	
5	152.58	
6	131.86	
7	158.57	
8	91.41	6.71 (s)
9	152.10	
10	105.05	
1′	121.48	
2′	113.52	7.43 (d, 1.9)
3′	149.82	
4′	145.79	
5′	115.96	6.9 (d, 8.9)
6′	119.03	7.45 (dd, 1.95, 7.8)
3′-OCH_3_	56.40	3.73 (s)
6-OCH_3_	60.01	3.91 (s)
OH-5		12.94 (s)
OH-7		

## Data Availability

The raw data supporting the conclusions of this article will be made available by the authors on request.

## Supplementary Materials

The following supporting information can be downloaded at https://www.mdpi.com/article/10.3390/molecules31183180/s1, Figure S1: Mass spectrum of (a) agarospirol (1) and (b) rosifoliol (2); Figure S2: HPLC chromatogram and UV spectrum of ursolic acid (3); Figure S3: Mass spectrum (ESI) of ursolic acid (3); Figure S4: Mass spectrum (ESI) of 7α-hydroxysandaracopimaric acid (4); Figure S5: ^1^H-NMR (CD_3_COCD_3_, 400 MHz) of 7α-hydroxysandaracopimaric acid (4); Figure S6: ^13^C-NMR (CD_3_COCD_3_, 100 MHz) of 7α-hydroxysandaracopimaric acid (4); Figure S7: ^1^H-^1^H COSY NMR (CD_3_COCD_3_, 400 MHz) of 7α-hydroxysandaracopimaric acid (4); Figure S8: ^1^H-^1^H NOESY NMR (CD_3_COCD_3_, 400 MHz) of 7α-hydroxysandaracopimaric acid (4); Figure S9: ^1^H-^13^C HSQC NMR (CD_3_COCD_3_, 400 MHz) of 7α-hydroxysandaracopimaric acid (4); Figure S10: ^1^H-^13^C HMBC NMR (CD_3_COCD_3_, 400 MHz) of 7α-hydroxysandaracopimaric acid (4); Figure S11: ^1^H-NMR (DMSO-d_6_, 400 MHz) of jaceosidin (5); Figure S12: ^13^C-NMR (DMSO-d_6_, 100 MHz) of jaceosidin (5).

## Appendix A

To establish the prediction of binding between the ligands derived from the compounds isolated from *S. elegans*, the SwissTargetPrediction tool was used. This platform allows for the prediction of pharmacological targets and the binding affinities of ligands isolated from *S. elegans* extracts. When applying this predictive tool, the isolated molecules or ligands from the extracts are compared within a database of known compounds that have already been experimentally tested. The approach is based on a fundamental principle as follows: if two molecules are structurally similar, they are likely to act on the same proteins. The similarity between the experimental ligands (*S. elegans*) and the reference ligands (proposed by the platform) is quantified through complementary procedures. First, 2D similarity is determined by using molecular chemical fingerprints (FP2). The similarity between FP2 fingerprints is calculated using the Tanimoto index, which ranges from 0 to 1, indicating the degree of structural matching. Values close to 1 indicate that the molecules are highly similar in their planar (2D) structure. In addition, 3D similarity is assessed using the Electroshape 5D (ES5D) method. This approach considers not only the molecular shape but also other properties such as spatial coordinates (atomic positions), partial electric charge, and lipophilic interactions. When applying ES5D, vectors are generated to define both the experimental and reference molecules, which are then compared using Manhattan distance; again, values close to 1 indicate high similarity. The calculated 2D and 3D similarity values are integrated into a multiple logistic regression model, meaning that a mathematical system was fitted to assign appropriate weights to the 2D and 3D similarities. The outcome is a combined score: if this value is greater than 0.5, there is a high probability that both molecules act on the same protein target. Thus, the combined use of 2D descriptors (structure) and 3D descriptors (shape and physicochemical properties) makes the predictive method robust and highly effective for identifying pharmacological targets with strong reliability.
