# Supplementary material for: Effect of Rosifoliol/Agarospirol and 7α-Hydroxysandaracopimaric Acid from Salvia elegans Vahl on Local Inflammation and LPS-Activated RAW-Blue Cells
Source: Molecules. 2026 Sep 10;31(18):3180. doi: 10.3390/molecules31183180 (PMC13609771; doi:10.3390/molecules31183180)
Supplement: Supplementary file 1 [file molecules-31-03180-s001.zip › molecules-4545617-supplementary.pdf]

Article

Effect of Rosifoliol/Agarospirol and 7α-Hydroxysandaracopimaric Acid from *Salvia elegans* Vahl on Local Inflammation and LPS-Activated RAW-Blue Cells

a)

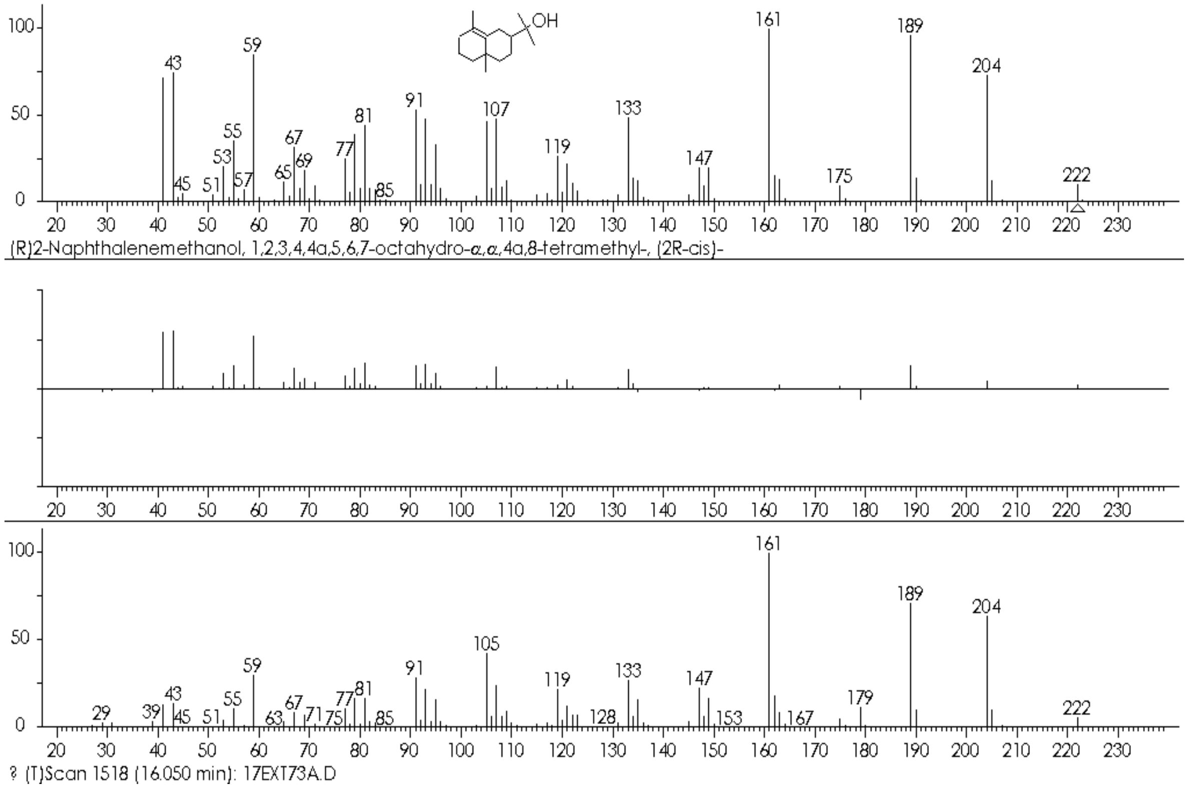

b)

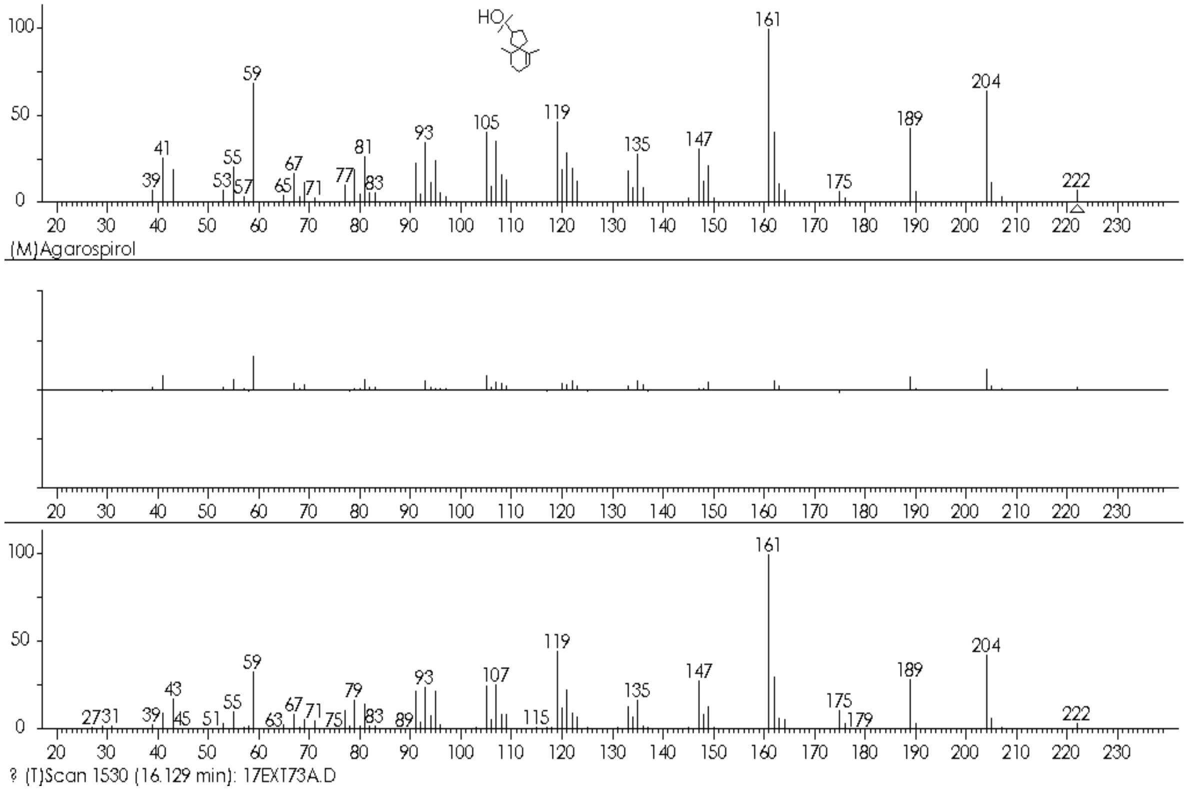

Figure S1. Mass spectrum of a) agarospirol (**1**) and b) rosifoliol (**2**)

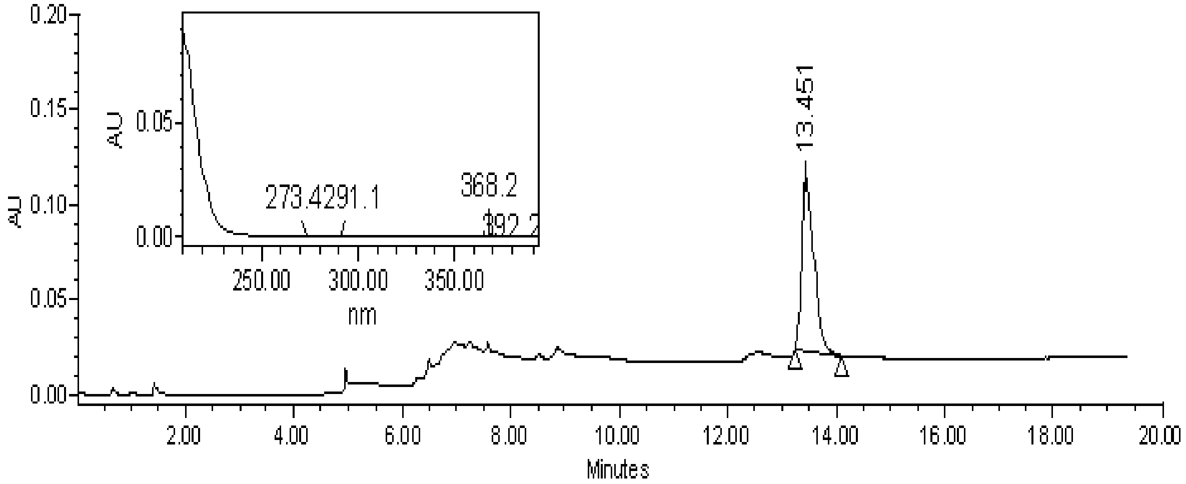

Figure S2. HPLC chromatogram and UV spectrum of ursolic acid (**3**)

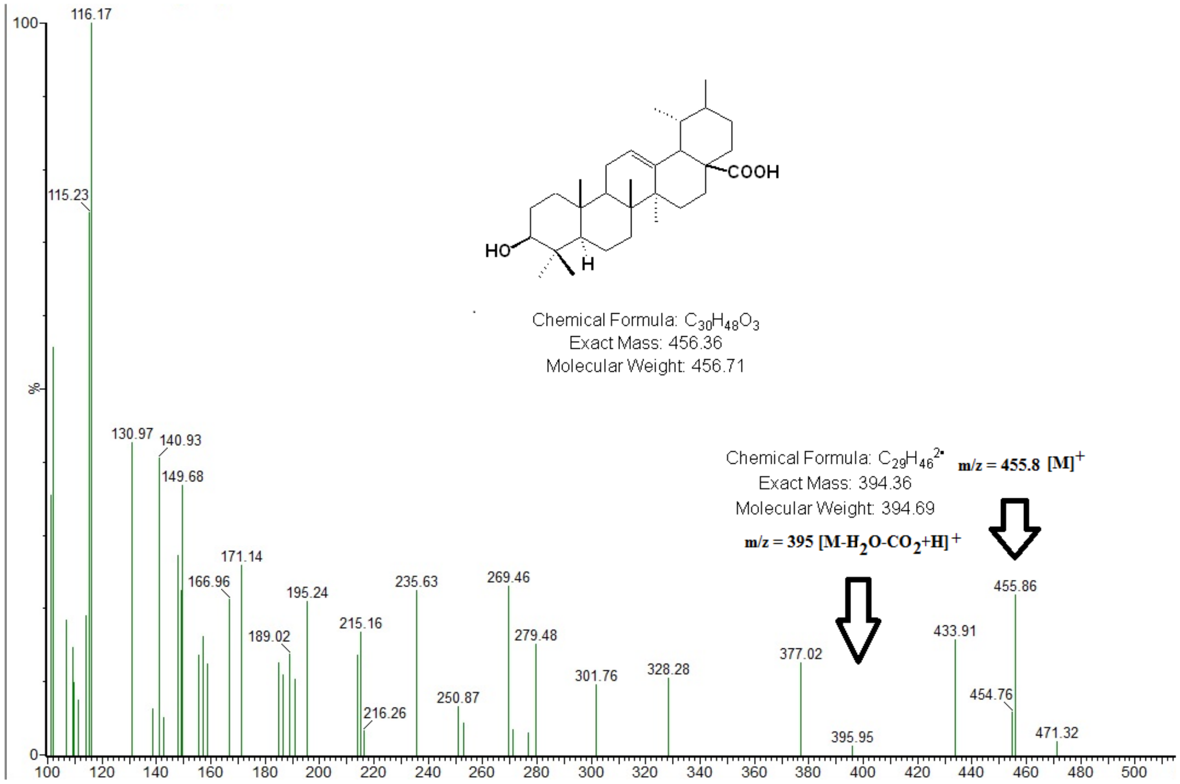

Figure S3. Mass spectrum (ESI) of ursolic acid (**3**)

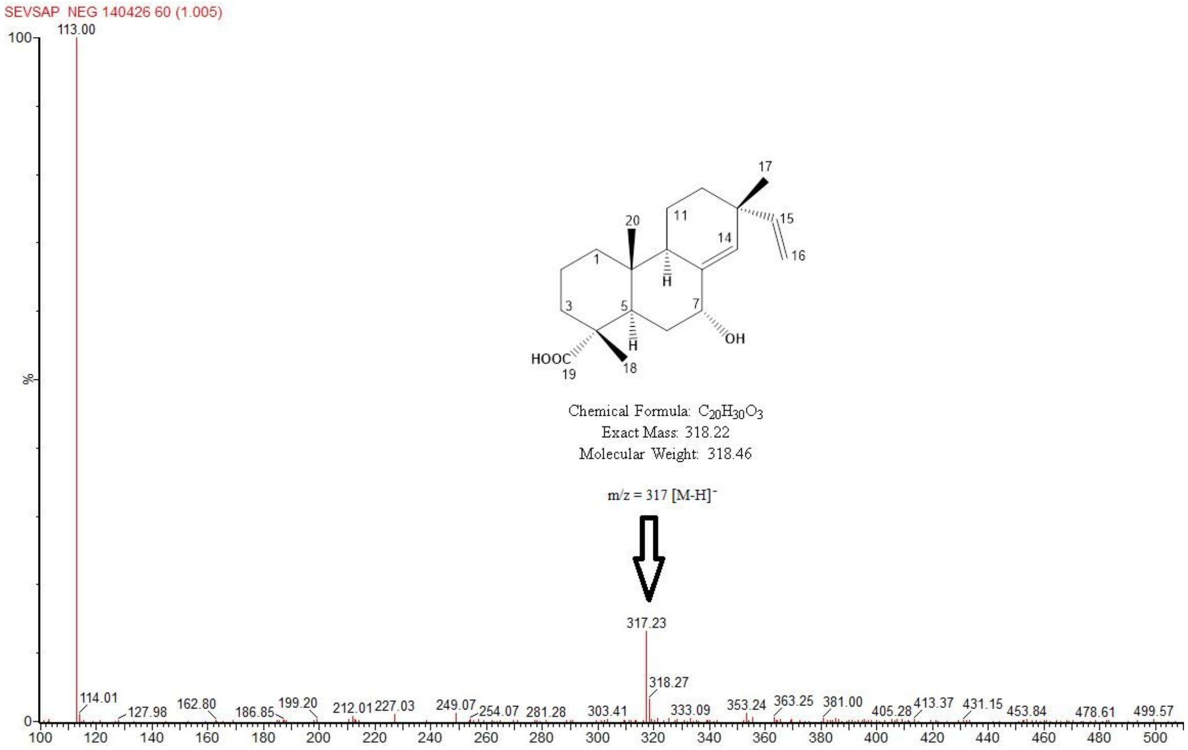

Figure S4. Mass spectrum (ESI) of 7α-hydroxysandaracopimaric acid (**4**)

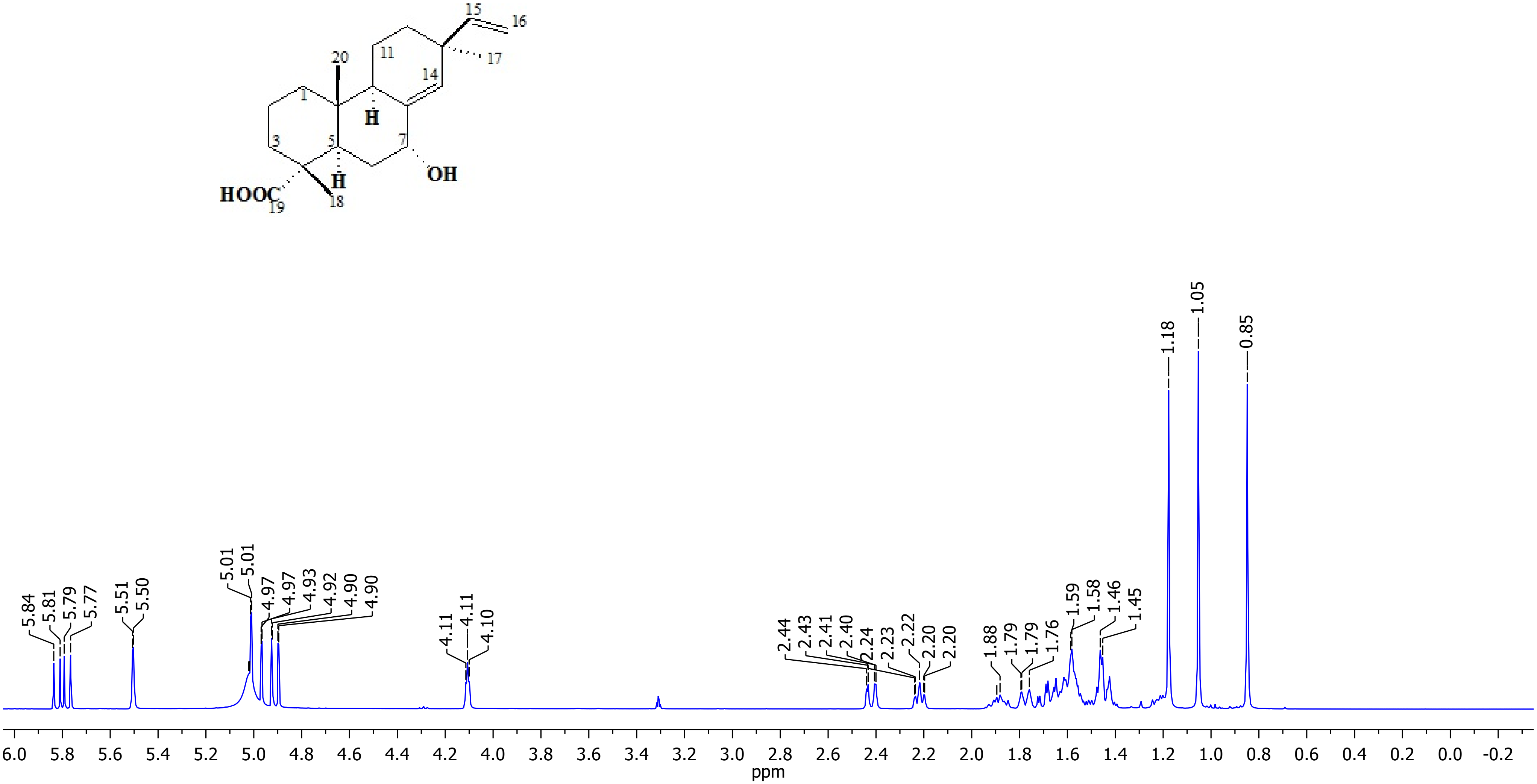

Figure S5 . ^{1}H-NMR (CD_3COCD_3, 400 MHz) of 7α-hydroxysandaracopimaric acid (**4**)

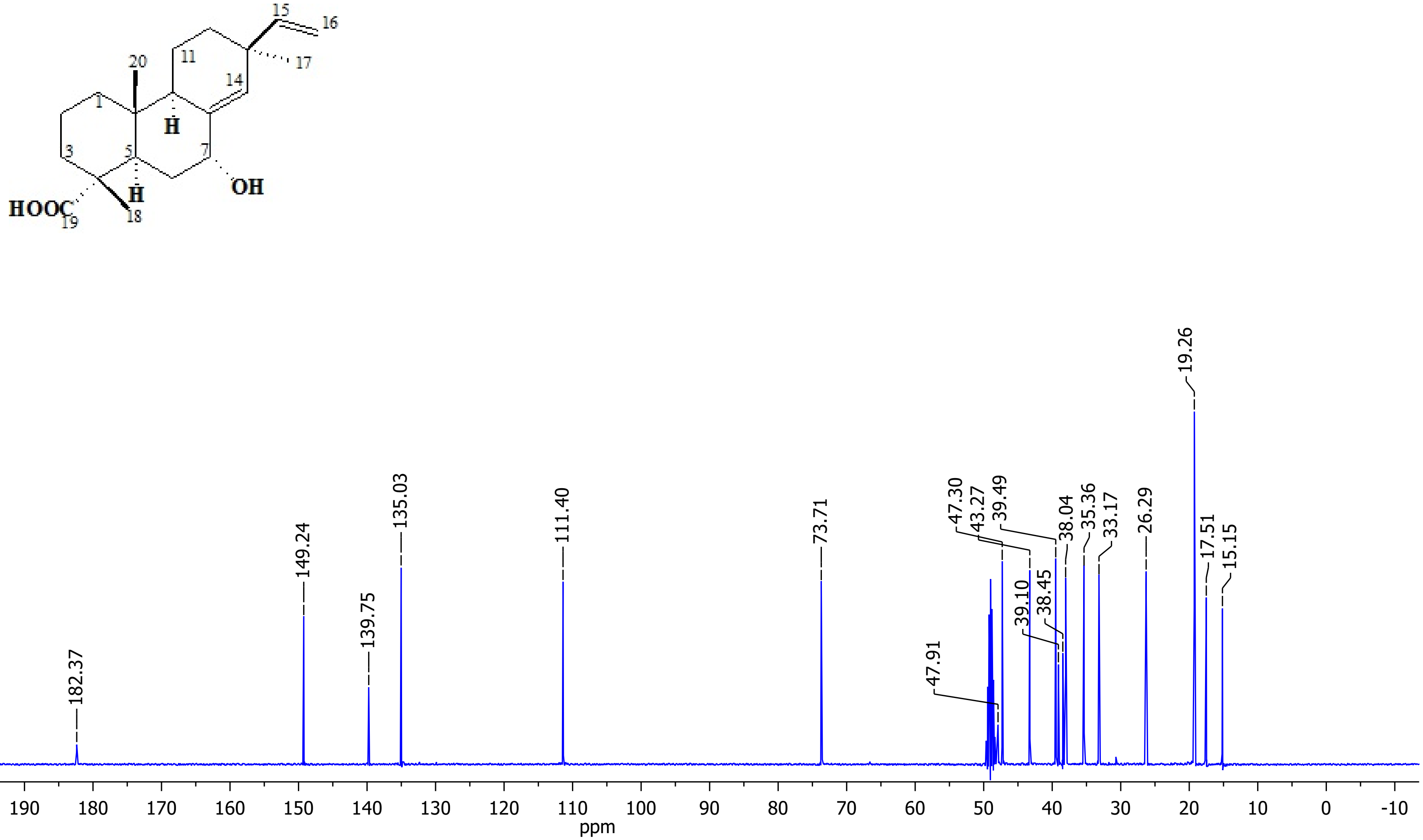

Figure S6 . ^{13}C-NMR (CD_3COCD_3, 100 MHz) of 7α-hydroxysandaracopimaric acid (**4**)

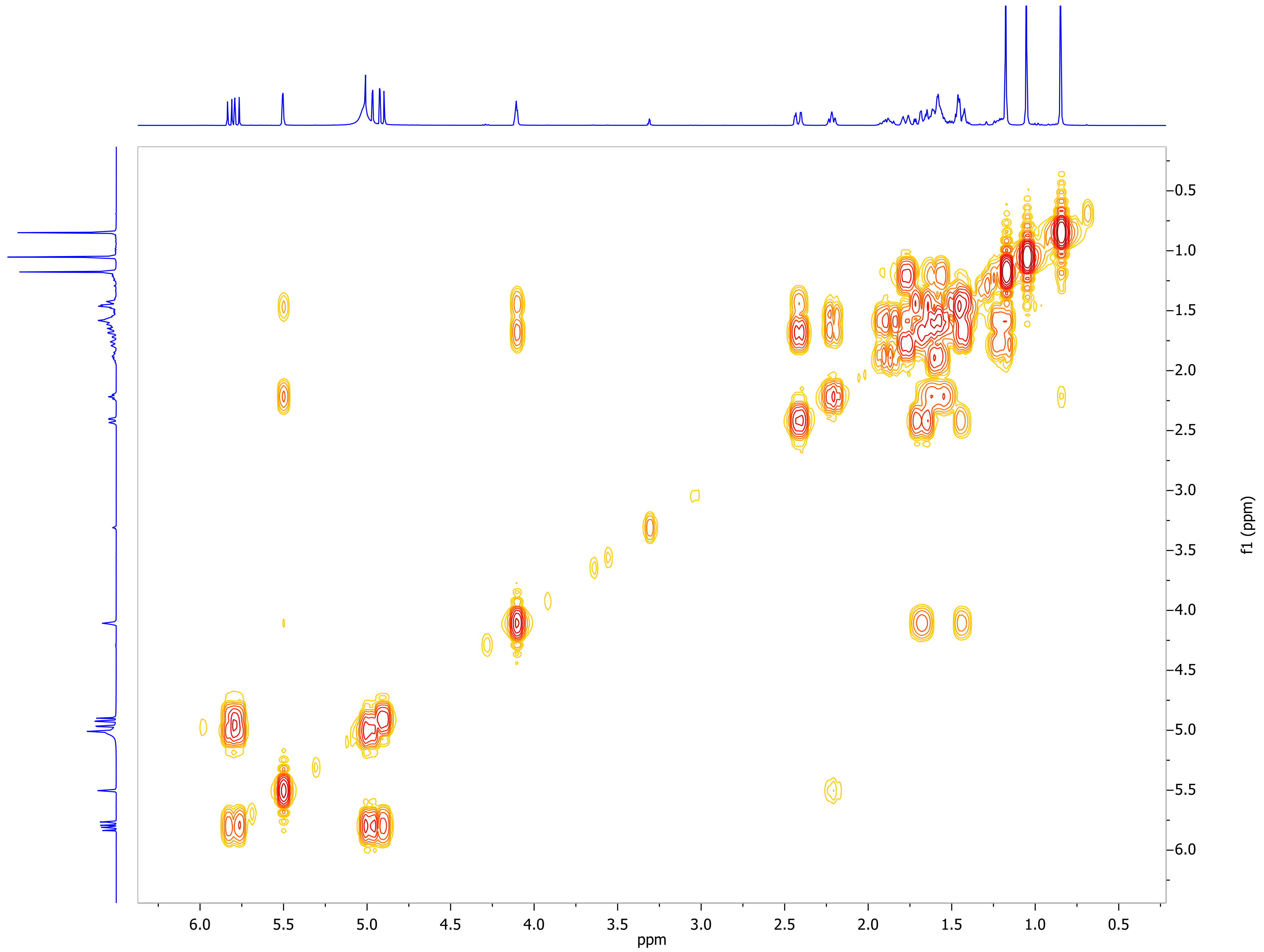

Figure S7 . ^{1}H-^{1}H COSY NMR (CD_3COCD_3, 400 MHz) of 7α-hydroxysandaracopimaric acid (**4**)

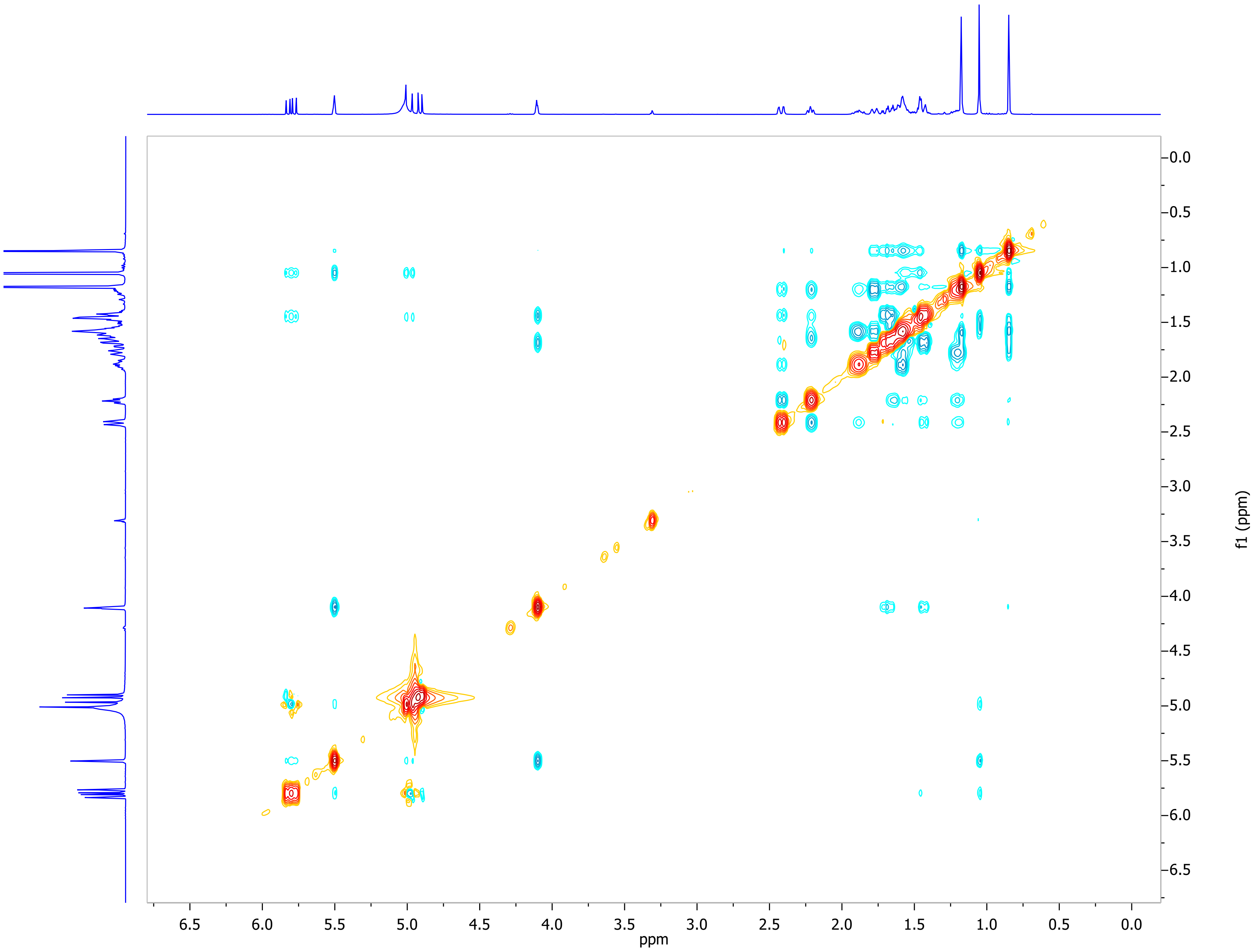

Figure S8 . ^{1}H-^{1}H NOESY NMR (CD_3COCD_3, 400 MHz) of 7α-hydroxysandaracopimaric acid (**4**)

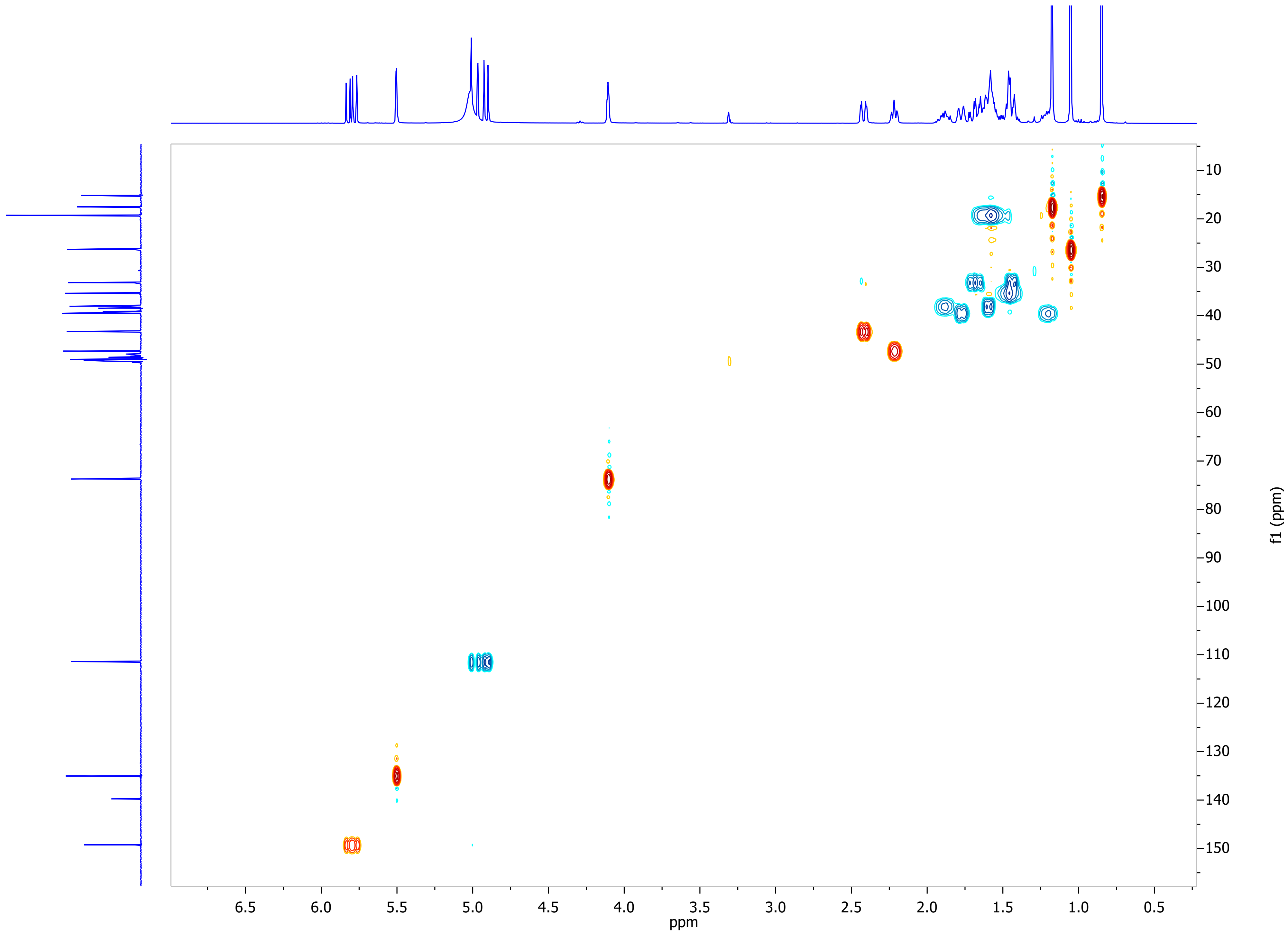

Figure S9 . ^{1}H-^{13}C HSQC NMR (CD_3COCD_3, 400 MHz) of 7α-hydroxysandaracopimaric acid (**4**)

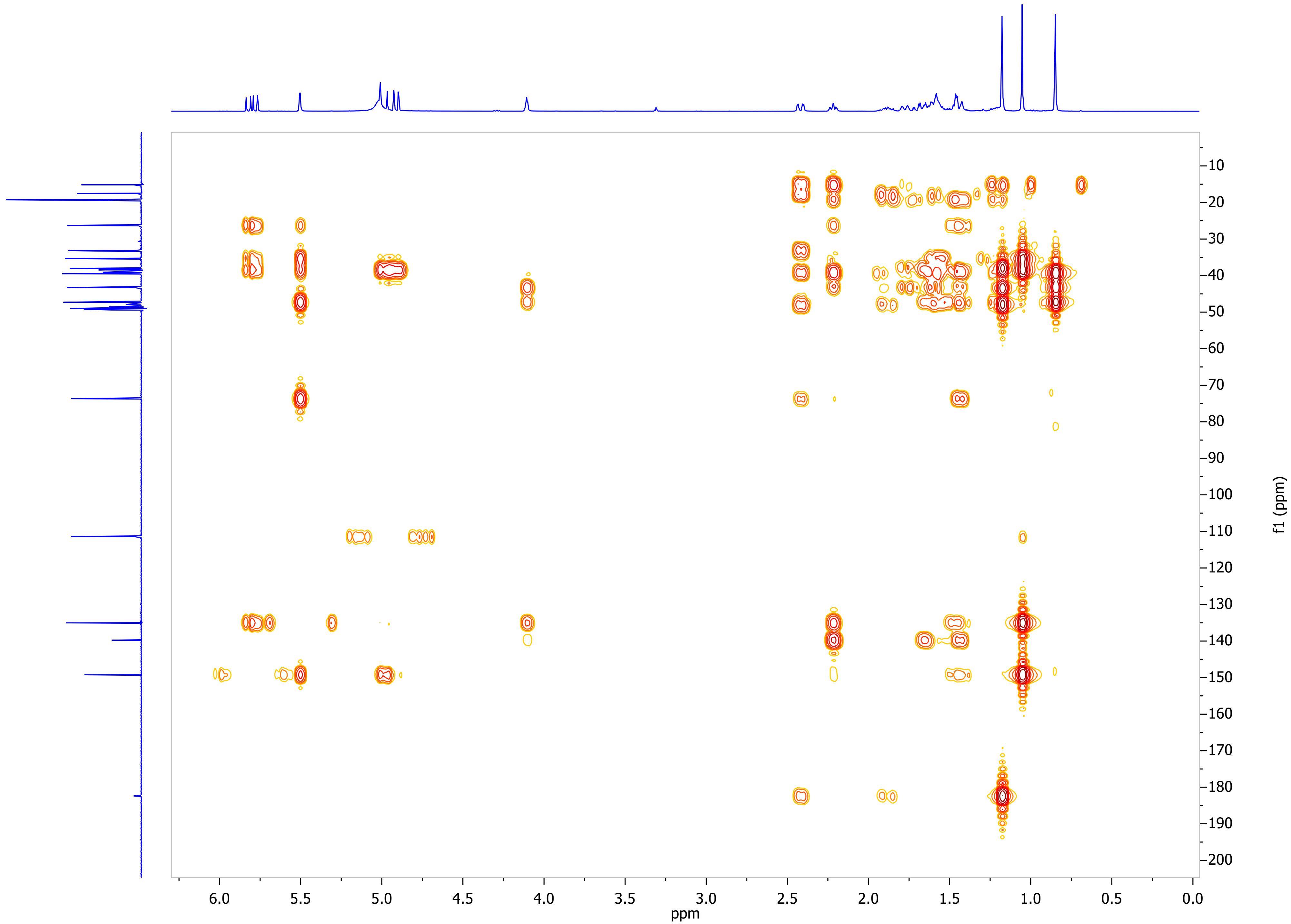

Figure S10 . ^{1}H-^{13}C HMBC NMR (CD_3COCD_3, 400 MHz) of 7α-hydroxysandaracopimaric acid (**4**)

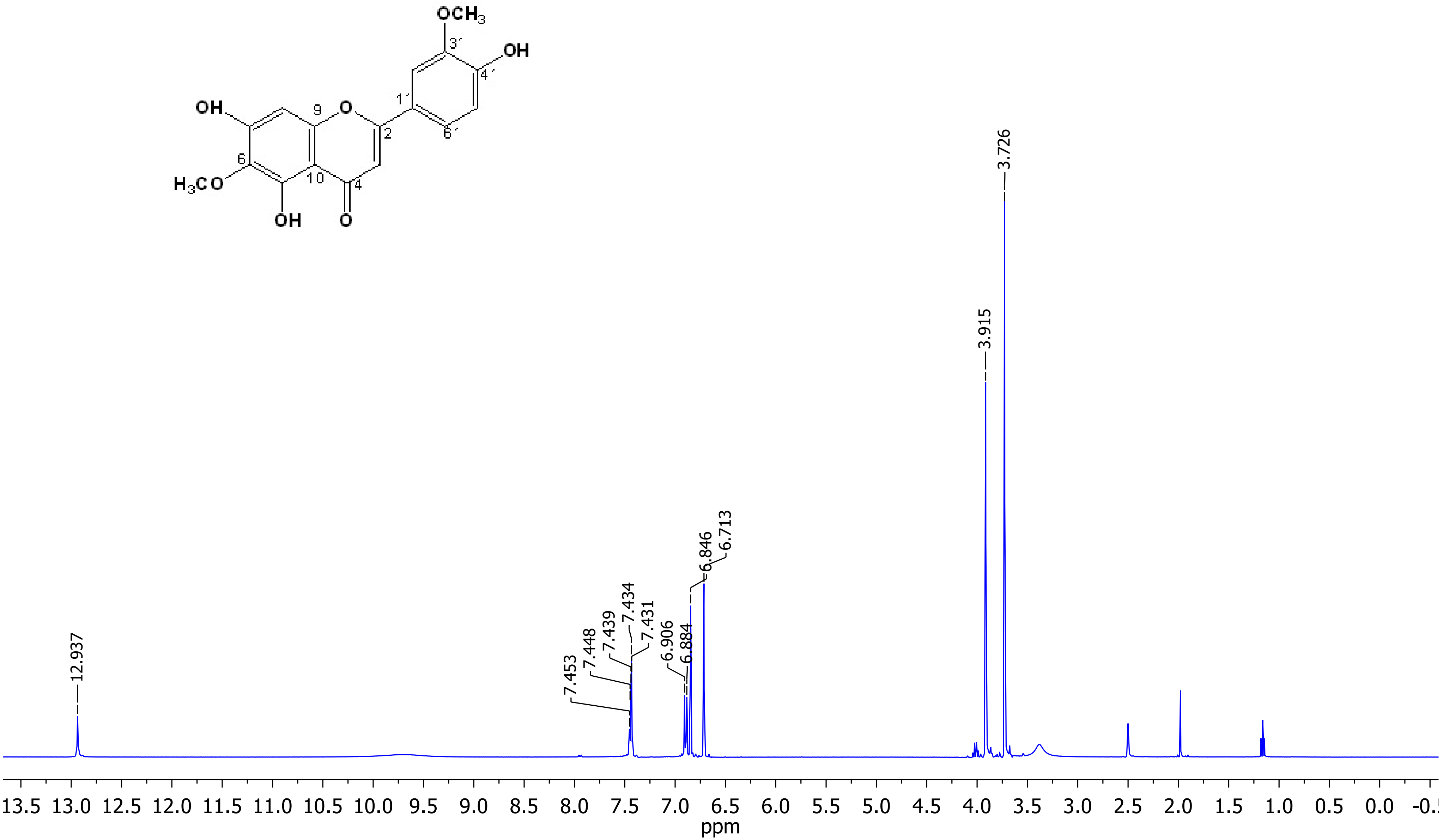

Figure S11. ^{1}H-NMR (DMSO-d_6, 400 MHz) of jaceosidin (**5**)

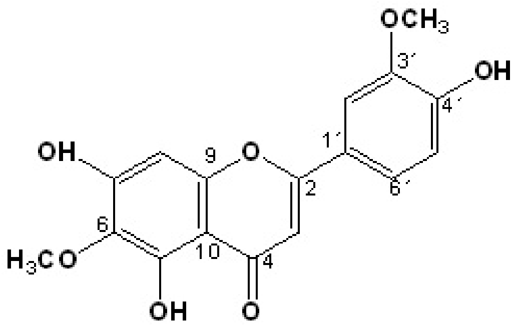

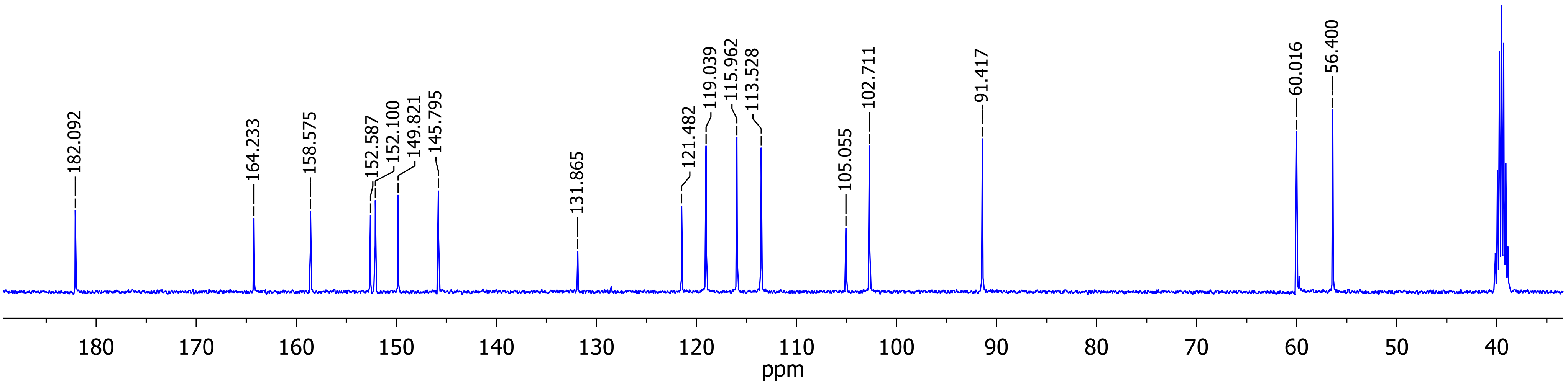

Figure S12. ^{13}C-NMR (DMSO-d_6, 100 MHz) of jaceosidin (**5**)
